# Specific contribution of cognitive and motor impairments with functional capacity and dependence in Huntington’s disease

**DOI:** 10.1007/s00415-025-12982-9

**Published:** 2025-02-22

**Authors:** Andres Gil-Salcedo, Marine Lunven, Charlotte Jacquemot, Renaud Massart, Anne-Catherine Bachoud-Levi

**Affiliations:** 1 Département d’Études Cognitives, École Normale Supérieure, PSL University, 75005 Paris, France; 2 Faculté de Médecine, Département Des Études Cognitives, Université Paris-Est Créteil, IMRB, Inserm U955, Ecole Normal Supérieure, NeuroPsychologie Interventionnelle, 29 Rue d’Ulm, 75005 Paris, Créteil, France; 3 Inserm U955, Institut Mondor de Recherche Biomédicale, Équipe NeuroPsychologie Interventionnelle, Créteil, France; 4 NeurATRIS, Mondor Node, Créteil, France; 5 APHP, Hôpital Henri Mondor, Centre National de Référence Maladie de Huntington, Service de Neurologie, Créteil, France

**Keywords:** Huntinton's Disease, Functional capacity, Dependency, Cognitive and Motor impairment, Autonomy loss

## Abstract

**Background:**

Huntington’s disease (HD) leads to increasing dependence. Unlike psychiatric disorders, motor and cognitive deficits evolve progressively over time. Understanding their specific impact on daily activities is crucial for preserving autonomy. However, because cognitive tasks in HD rely on motor functions, and motor tasks demand cognitive processing, disentangling their specific impact remains a challenge.

**Objective:**

To identify the specific contribution of cognitive and motor impairments on global functional capacity, basic and instrumental activities of daily living (ADL/IADL), and work-related activities (WRA) in HD.

**Methods:**

158 HD mutation carriers, enrolled in the BioHD (NCT01412125) and RepairHD (NCT03119246) studies, were evaluated with the Unified Huntington’s Disease Rating Scale and the SelfCog. The SelfCog assesses motor processing separately from memory, language, executive functions and visuospatial processing. Linear regressions were fitted to assess how functional capacity declined with motor and cognition impairments. Odds of dependence in ADLs, IADLs and WRAs were estimated using logistic regressions.

**Results:**

Cognitive and motor performance were independently associated with functional capacities, though motor performance showed a stronger association than cognitive performance. Decline of all SelfCog cognitive domains contributed to functional decline, with stronger association with global and executive scores compared to language, visuospatial, and memory domains. Higher global and executive deficits were associated with an increased risk of dependence in ADLs, IADLs, and WRAs.

**Conclusion:**

The independent contributions of motor, followed by cognitive—mainly executive—functions to functional decline suggest targeted interventions to preserve autonomy and quality of life in HD.

**Supplementary Information:**

The online version contains supplementary material available at 10.1007/s00415-025-12982-9.

## Introduction

Huntington’s disease (HD) is an inherited neurodegenerative disorder characterized by progressive motor, and cognitive impairments [1, 2], as well as psychiatric manifestations [3]. The clinical onset usually occurs after the age of 30 [1, 4]. Functional decline begins in the early stages [5] and affects all patients [6]. Individuals experience limitations in work-related activities (WRAs, maintaining employment) [7] as well as in executing basic and instrumental activities of daily living (ADLs and IADLs) [8–10]. This Functional decline accelerates as motor and cognitive symptoms [11] increase until patients reach a high level of dependence [5, 12, 13] eventually requiring full care or institutionalization [9, 14].

Due to the impact of Huntington’s disease (HD) symptoms on functional capacity, and the fact that WRAs, ADLs, and IADLs are now regarded as health indicators in both chronic diseases and aging populations [15–17], regulatory agencies such as the FDA and the European Medicines Agency require that interventions demonstrate not only motor or cognitive improvements but also functional benefits [18, 19]. To enhance patient care and develop personalized therapeutic interventions that preserve autonomy and quality of life, it is thus essential to understand the specific impact of cognitive and motor decline on functional scores and daily activities. However, distinguishing the individual contributions of cognitive and motor impairments to functional limitations [8, 20] remains challenging.

Better cognitive and motor performance is associated with better functional scores as well as a slower rate of decline [6, 8, 13, 20] on the three specific scales from the Unified Huntington Disease Rating Scale (UHDRS): the Total Functional Capacity (TFC) [21], the Independence Scale (IS), and the Functional Checklist Score (FCS) [21]. Regarding the daily activities, cognitive deficits are primarily associated with WRAs and work cessation, followed by motor impairments [7, 8]. Poor global cognitive performance and slow reaction time are associated with the loss of IADLs, such as driving autonomy [22, 23], as well as cognitive impairment and apathy correlate with difficulties in managing finances [24]. Both cognitive and motor factors are strongly associated with ADLs, such as self-care dependence [25] and institutionalization [10, 14, 26]. Yet, because the motor and cognitive tasks are not specific but often overlap, disentangling the contribution of cognitive and motor impairments to functional limitations [8, 20] remains challenging.

Indeed, the cognitive tasks included in the UHDRS (Stroop, Symbol Digit modality Task, and Literal Fluency) and the worldwide Enroll-HD platform (Category Fluency and Mini-mental state examination/Montreal cognitive assessment), although robust markers of cognitive evolution in HD [27, 28], require written responses or timed verbalization that involve motor functions [20, 29]. Likewise, the Total Motor Score (TMS) of the UHDRS includes tasks that involve cognitive processing, such as attention, executive functions, and working memory (e.g., Luria sequences). Besides, differences in task procedures (e.g., different time constraints, accuracy calculation, different executive load, etc.) make precise comparisons across cognitive domains impossible [30].

Here, we aimed to identify the respective contribution of cognitive and motor impairments on functional capacities, as well as ADLs, IADLs and WRAs dependence, in HD mutation carriers. We focused principally on patients with slight cognitive changes, often targeted in therapeutic trials. To evaluate the contribution of motor and cognitive domains separately (executive, visuo-spatial, memory, and language performance), we used the SelfCog, a digital cognitive battery that allows sensitive assessment of four cognitive domains (executive, visuo-spatial, memory, and language) independent of motor capacity [31]. This test has shown high sensitivity and specificity even on patients with mild functional impairment, although their cognitive impairment is often mild and rather difficult to specify [29, 32]. Second, in order to assess daily living situations, as ADLs, IADLs and WRAs in HD mutation carriers, we grouped the FCS items according to the type of activity.

## Methods

### Study population

Data were extracted from the BioHD (NCT01412125) [33] and RepairHD (NCT03119246) [34] prospective longitudinal observational studies approved by French Ethical Committees (CPP). Participants were HD mutation carriers, confirmed by genetic testing cytosine–adenine–guanine (CAG) expansion (≥ 36 CAG repeats), over 18 years of age, having signed an informed consent form. Inclusion in these analyses required functional capacity data (TFC, IS, and FCS), SelfCog assessment, and at least three of the five UHDRS cognitive tasks completion. Missing UHDRS cognitive scores were imputed with missForest [35] (see Supplementary Methods). For participants who completed multiple evaluations, visits with the lowest TFC, IS and FCS scores were retained for analysis.

### Functional capacity

Functional capacity was assessed using the UHDRS (TFC, IS, and FCS) [21, 36]. The TFC ranges from 0 to 13, with 13 being normal and 0 being a complete loss of capacity. IS score range from 0 to 100, 0 representing no need and 100 indicating bedridden/tube fed. The FCS consists of 25 items questioning the patient and if available his/her proxy on the ability to perform an activity (e.g., “Could subject dress himself/herself without help?”). A score of 25 signifies full functional capacity, while 0 means a need for assistance in all activities. FCS scores on specific items were used to establish the dependence status on ADLs, IADLs and WRAs. The selection and grouping of ADLs, IADLs, and WRAs, as well as their level of difficulty and internal consistency, was assessed using factor analysis and RASCH analysis (see Supplementary Methods). Dependence was considered for a whole group of activities if any of the items in the group could not be performed without assistance.

### Cognitive impairments and motor deficiencies

Cognitive UHDRS tests were the Symbol Digit Modalities Test (SDMT), Stroop tests (colour, word and interference), and the Literal Fluency test [21]. A high score (number of correct answers) on these tests indicates good cognitive performance.

The SelfCog is a brief yet comprehensive digitized cognitive battery that takes approximately 15 min to complete. It is designed to assess motor, executive, visuospatial, language and memory functions, along with motor performance in 5 comparable tasks by 40 trials each. It can be administered by non-experts with minimal training and offers a standardized approach, allowing to compare performance in each cognitive domain. Participants respond by pressing as quickly as possible one of two buttons while looking at pairs of images, with task instructions varying for each domain. The motor task measures the time needed to process and respond to a simple visual stimulus and establishes the baseline response time which is subtracted from the reaction times of cognitive tasks. The visuospatial subtest evaluates visuospatial skills through trials involving either visually different exemplars of the same object involving, mental rotation, or semantically unrelated but visually similar objects, or semantically related but visually dissimilar objects. The language subtest evaluates naming skills by asking the participant to decide whether the two pictures begin with the same syllable or not. The executive function task involves managing dual tasks and contrasts trials requiring task-switching with those that do not. The memory task is the final subtest and evaluates the memory ability. The participant has to decide between the two images, which one was presented previously. Short-term memory is assessed using images repeated within the memory subtest, while long-term memory is assessed through the recognition of images previously encountered in the subtests. All instructions are presented in Lunven et al. (2023) [31]. At the end of the assessment, the tool provides automatic sub-scores for each function, along with a composite score, helping to identify affected cognitive functions and enabling targeted interventions and informed clinical decisions. The inverted efficiency score (IES), calculated as IES = correct RT/Accuracy, balances the trade-off between speed and accuracy. It accounts for the tendency of slower responses to correlate with lower error rates and faster responses with higher error rates [37]. A higher IES reflects poorer performance, while a lower IES, close to 1, indicates better performance.

Motor impairments were assessed with the SelfCog motor IES and the Total Motor Score (TMS) of the UHDRS. Unlike motor IES, a high TMS score indicates high motor impairment (from 0 to 124).

### Descriptive statistical analysis

First, sociodemographic data and clinical scores of participants with (TFC < 13) and without functional limitations (TFC = 13) were compared using Pearson's chi-square test for categorical variables, and a t-test for continuous variables. Second, similarly, we compared the average IES of participants with or without limitations for each of the daily activities, using either a t-test or a Wilcoxon–Mann–Whitney test.

### Association between cognitive performance and functional limitation scores

The association of cognitive performance and motor impairments with functional limitation was evaluated by estimating multivariate linear models. In these models, each functional limitation scale was used as the dependent variable (TFC, IS and FCS), while cognitive and motor scores were used as the explanatory variable (global, executive, visual, language, memory, motor SelfCog IES and each UHDRS cognitive score and TMS). Three-stage model adjustments were performed to estimate the specific contributions of cognitive and motor deficits to functional impairments. Type 1 (T1) models correspond to univariate analysis of both cognitive and motor tests without adjustment. Type 2 (T2) models correspond to an analysis of each cognitive test adjusted for each motor test (Motor IES and TMS). Type 3 (T3) models correspond to an analysis of each cognitive test adjusted for each motor test, in addition to age at visit and years of education. To facilitate the comparison of the results between the different cognitive tests, the models were also replicated with the IES scores normalized to the mean and the standard deviation ([IES-Mean_score]/SD) and the UHDRS test scores inverted and normalized to the mean and the standard deviation (([Score-Mean_score]/SD)*(− 1)). Additionally, in the fully adjusted models (type 3 models), the variance inflation factor (VIF) was evaluated to identify potential multicollinearities (a VIF close to 1 indicates low multicollinearity between variables), as well as the tolerance (percentage of variance in the predictor that cannot be explained by other predictors) to determine the individual contribution of each cognitive and motor factor in explaining the variance of the functional tests.

### Association between cognitive performance and dependence in ADL, IADL and WRA

Associations between ADLs, IADLs and WRAs dependence and cognitive IES were evaluated by a logistic model adjusting for age at visit, years of education, and motor IES. The same approach was used for specific items of IADLs (Transport, Driving, Finances, Grocery shopping and Housekeeping). All analyses were conducted in R 4.2.2. The data that support the findings of this study are available from the corresponding author upon reasonable request.

### Post hoc* analysis*

While neuropsychiatric symptoms do not generally exhibit a linear association with HD progression [38], they may influence cognitive, motor, and functional impairment at all stages of the disease. Accordingly, the full adjusted model (type 3) was replicated by separately including Problem Behaviour Assessment-short (PBA-s) [39] domain scores for apathy, depression, irritability, psychosis, and obsessive–compulsive disorders [38, 40]. Exposure to treatments with antidepressants, anxiolytics, antipsychotics, and other neurological medications (e.g., anticonvulsants) was also included to assess the stability of the specific contributions observed in the main outcomes. Furthermore, different phenotypes with motor, cognitive, or mixed predominance may be present during the progression of HD [41, 42]. To identify potential differences in phenotypes in our population, a clustering (k-means) analysis was conducted incorporating all cognitive assessments and the specific items of the TMS. The potential interactions between the study variables and the phenotype groups in the fully adjusted model were then evaluated.

### Statistical sensitivity analysis

To assess potential biases due to the difficulty identifying temporal variability in a cross-sectional analysis [43], we calibrated linear mixed models with a Bayesian approach in 92 individuals who had multiple visits during follow-up. We also replicated the analyses with normalized cognitive tests by adjusting for the TMS instead of the motor IES to address a possible underestimation of the motor component. Given the absence of participants with severe functional deficits (TFC = 10.5(SD = 2.4); Q1 = 9.0, Q3 = 13) that may result in an underestimation of their association with cognitive impairment, we replicated the analysis by including a propensity score estimated as the probability of having a specific IES as a function of age, education, sex, CAG repetitions, and gender [44]. Finally, to confirm the robustness of the results without the imputed data, we replicated the analyses excluding the 14 individuals for whom some UHDRS cognitive scores were imputed.

## Results

**Fig. 1:**
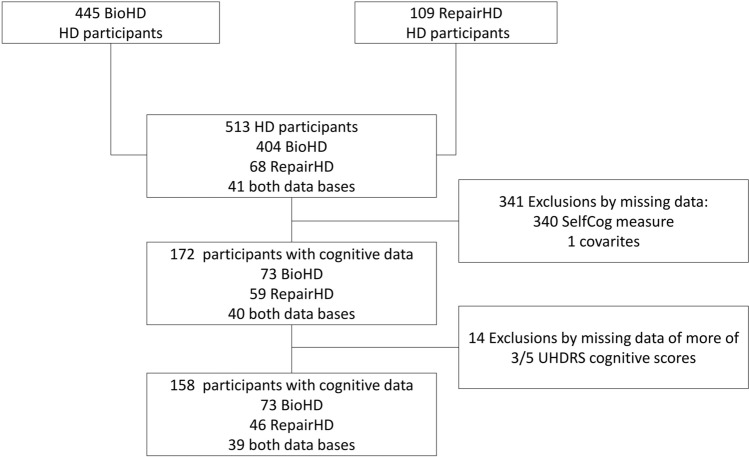
Flowchart of the population selection

Out of 513 HD participants extracted from the BioHD and RepairHD studies, 158 were maintained in the analyses (Fig. 1). In 14 participants, missing data for up to 3 UHDRS cognitive tests were imputed.

Population characteristics are displayed in Table 1. Participants with TFC < 13 (mean = 9.70, SD = 2.00) were 65.8% and those with TFC at 13 were 34.2% with no difference in sex, years of education or number of CAG repeats between the two groups (*p* > 0.064 for all). Functionally impaired participants were older and had poorer motor and cognitive performance (*p* < 0.001 for all).

**Table 1:** Population characteristics

Characteristics	Total (N = 158)	TFC < 13 (n = 104)	TFC = 13 (n = 54)	p-value
Sex				0.083
Male	91 (57.6%)	65 (62.5%)	26 (48.1%)	
Female	67 (42.4%)	39 (37.5%)	28 (51.9%)	
Age				< 0.001
Mean (SD)	50.26 (11.71)	53.50 (10.42)	44.03 (11.61)	
Years of study				0.064
Mean (SD)	14.06 (3.04)	13.74 (3.31)	14.69 (2.35)	
CAG repeats				0.216
Mean (SD)	43.18 (3.05)	43.39 (3.29)	42.76 (2.51)	
Disease Burden Score				< 0.001
Mean (SD)	366.57 (103.03)	398.87 (94.18)	304.35 (90.65)	
SelfCog IES, Mean (SD)				
Global	2.88 (1.40)	3.33 (1.47)	2.01 (0.66)	< 0.001
Executive	1.42 (0.85)	1.65 (0.91)	0.99 (0.48)	< 0.001
Visual	1.58 (1.11)	1.87 (1.24)	1.00 (0.35)	< 0.001
Language	6.08 (3.46)	7.11 (3.73)	4.09 (1.50)	< 0.001
Memory	2.45 (1.34)	2.70 (1.48)	1.97 (0.85)	< 0.001
Motor	0.73 (0.32)	0.84 (0.33)	0.51 (0.13)	< 0.001
UHDRS cognitive scores				
SDMT	36.51 (15.01)	29.33 (11.38)	50.33 (10.93)	< 0.001
Stroop Word	75.91 (24.19)	64.81 (18.41)	97.30 (19.16)	< 0.001
Stroop Colour	57.18 (18.44)	48.74 (14.11)	73.44 (14.53)	< 0.001
Stroop Interference	32.23 (12.43)	27.00 (9.42)	42.30 (11.34)	< 0.001
Literal fluency	30.69 (12.81)	25.82 (10.69)	40.07 (11.31)	< 0.001
Other Functional Limitation score				
Independence Scale	88.01 (11.65)	81.88 (9.76)	99.81 (0.95)	< 0.001
Functional Checklist Score	22.09 (3.57)	20.60 (3.57)	24.98 (0.14)	< 0.001
Total Motor Score	22.10 (19.18)	31.45 (16.72)	4.28 (7.11)	< 0.001

Mean (standard deviation) except where otherwise indicated

*TFC* Total functional Capacity, *CAG* cytosine–adenine–guanine, *SDMT* Symbol digit modality test

### Association between cognitive performance and functional limitation scores

Univariate models showed significant associations between functional capacity and motor scores [motor IES (TFC: coef. = − 3.46, IS: coef. =  − 19.38, FCS: coef. =  − 6.01); TMS (TFC: coef. =  − 0.09, IS: coef. = −0.48, FCS: coef. =  − 0.13); *p* < 0.001 for all; Tables S1–S2, T1 models). After adjustment with cognitive IES (T2 models) and full adjustment (T3 models), the associations remained significant, although slightly attenuated [motor IES (TFC: coef. =  − 2.53 to − 1.82, IS: coef. =  − 14.29 to − 9.76, FCS: coef. =  − 4.97 to − 3.59); TMS (TFC: coef. =  − 0.08 to − 0.08, IS: coef. − 0.44 to − 0.40, FCS: coef. =  − 0.13 to − 0.11); *p* ≤ 0.012 for all; Tables S1-S2; T3 models). After adjustment for UHDRS cognitive tests and demographics, the associations also remained significant with TMS (TFC: coef. =  − 0.08 to − 0.06, IS: coef. =  − 0.40 to − 0.33, FCS: coef. =  − 0.12 to − 0.11; *p* ≤ 0.012 for all; Table S2) but not with motor IES (TFC: coef. =  − 1.75 to − 0.59, IS: coef. =  − 9.35 to − 3.95, FCS: coef. =  − 4.11 to − 2.36; *p* > 0.05 for all; Table S1).

In fully adjusted linear models, greater multicollinearity was observed between motor and UHDRS cognitive scores (except for Verbal Fluency) (TMS, VIF = 2.11–2.68; motor IES, VIF = 1.33–1.96) than between motor scores and cognitive IES (TMS, VIF = 1.23–1.59; motor IES, VIF = 1.16–1.44). Multicollinearity was higher for cognitive scores with TMS than with motor IES. Consistently, motor scores were more correlated with the UHDRS cognitive scores (except for Verbal Fluency) (Pearson coefficients TMS = − 0.70 to − 0.78; motor IES = − 0.61 to − 0.63) than with cognitive IES (Pearson coefficients TMS = 0.41–0.60; motor IES = 0.35–0.53). Cognitive IES, adjusted for motor IES, had a higher explanatory power of variance in functional capacity, with a tolerance of 69 to 85%, than UHDRS cognitive tests, adjusted for motor IES, with a tolerance of 58–75%. Tolerance of all cognitive tests, adjusted for TMS, did not exceed 80%, with a minimum value of 37% for SDMT. Therefore, we considered that models adjusted for motor IES (Table S1–T3 models), and not for TMS, are the most suitable to distinguish motor from cognitive impacts on functional capacities while controlling for possible multicollinearity.

**Fig. 2:**
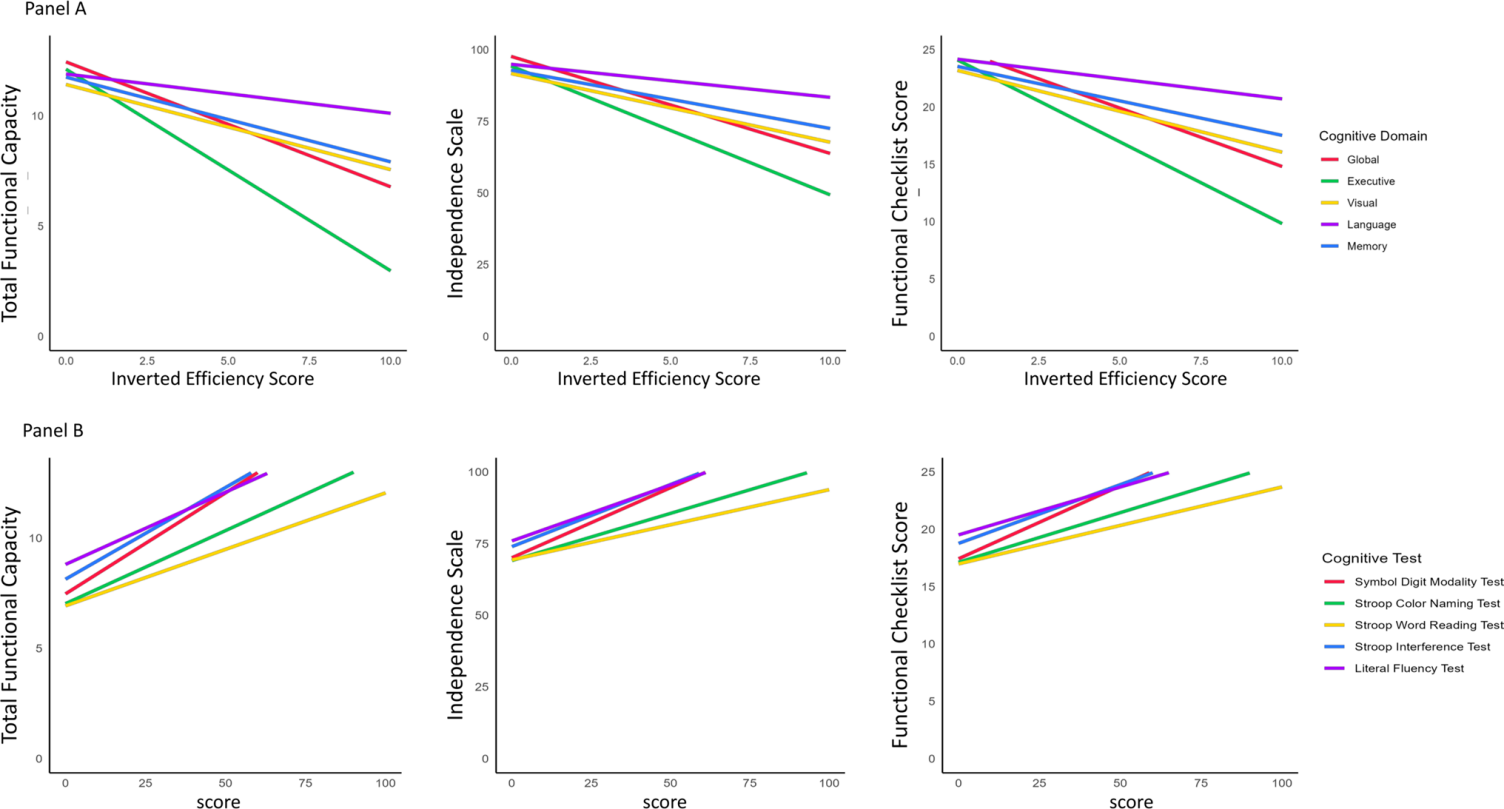
Association between cognitive and functional scores. Panel A: Association between the SelfCog IES and functional limitations scales. Panel B: Association between the UHDRS cognitive tests and functional limitations scales. Mean trajectories of linear models adjusted for age, education years and motor IES. Coefficients in Table S1-model 3. Results with scaled scores in Figure S1 and Table S3–T3 models

The univariate analyses revealed significant associations between cognitive and functional scores, even after adjustment for motor IES (*p* < 0.001 for all, Table S1, T1 vs. T3 models). Associations with functional scores were stronger for global IES (TFC: coef. =  − 0.57, IS: coef. =  − 3.39, FCS: coef. =  − 1.02; *p* < 0.001 for all) and executive IES (TFC: coef. =  − 0.92, IS: coef. =  − 4.50, FCS: coef. =  − 1.43; *p* < 0.001 for all) compared to visual, memory and language IES (details in Table S1 panel A). Regarding UHDRS scores, SDMT and Stroop Interference showed the strongest associations with functional scales (TFC: coef. = 0.09–0.08, IS: coef. = 0.49–0.43, FCS: coef. = 0.13–0.10, respectively; *p* < 0.001 for all, Table S1; Fig. 2 panel B).

The coefficients calculated with normalized scores (Figure S1, Table S3-T3 models) were higher, in most cases, for global (TFC = − 1.43, IS = − 8.55, FCS = − 2.57) and executive (TFC = − 2.31, IS = − 11.34, FCS = − 3.60) IES than for UHDRS cognitive tests (TFC = − 0.84 to − 1.38, IS = − 5.01 to − 7.34, FCS = − 1.07 to − 1.91).

### Association between cognitive performance and ADLs, IADLs, WRAs dependence

**Fig. 3:**
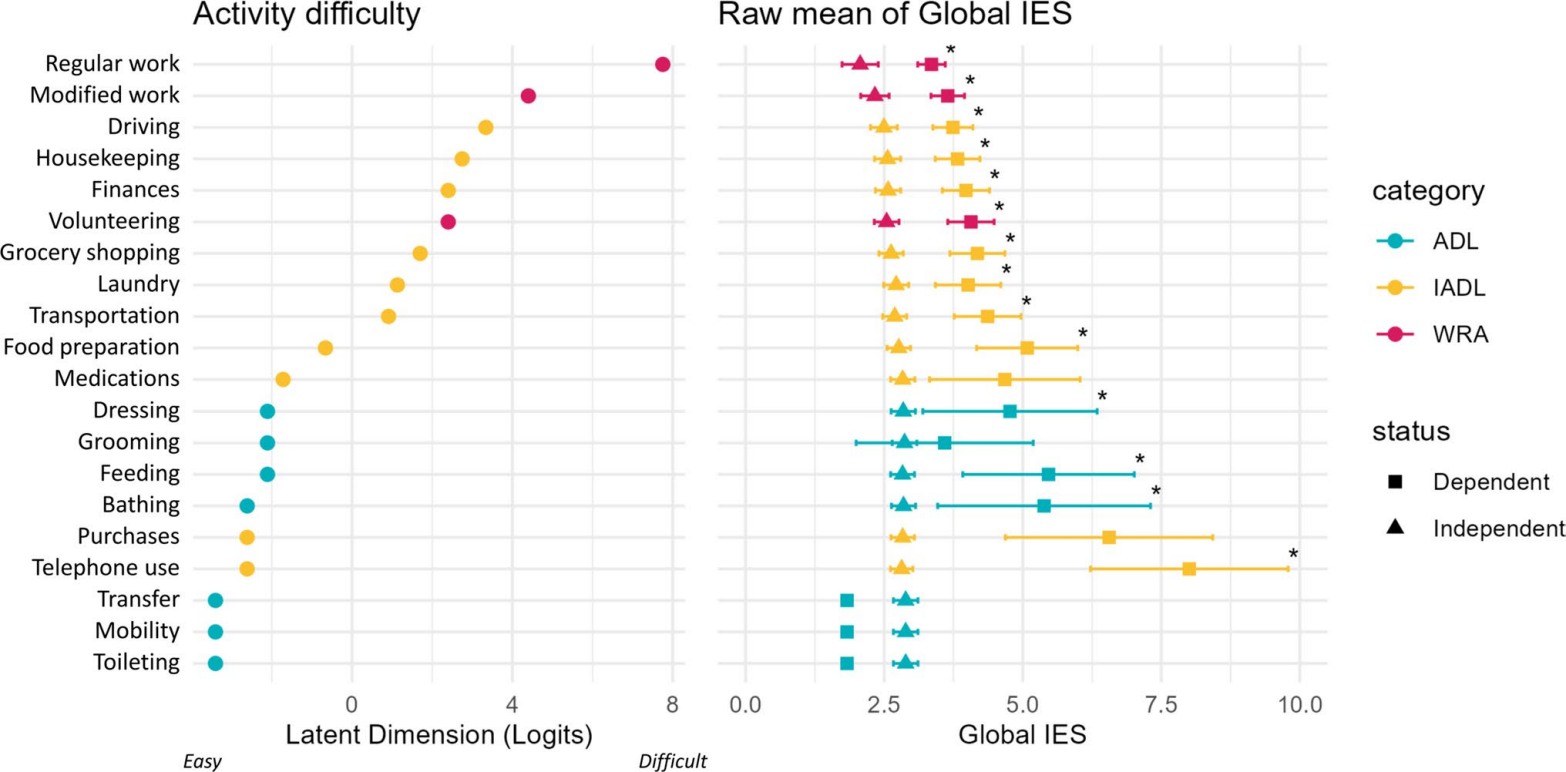
Item map difficulty and comparison of raw means of dependent and independent participants for each ADL, IADL and WRA. Left: RASCH analysis results. Category based on previous explore factor analysis and literature concepts (see Supplement Results). Right: Raw mean and confidence interval at 95%. ADL = activities of daily living; IADL = instrumental ADL; WRA = work-related activities, *significant mean difference (*p* value < 0.05). Dependence was considered if any ADL, IADL, or WRA could not be performed without assistance

The selection of the ADLs, IADLs, and WRAs was made using confirmatory factor and Rasch analyses, to ensure a consistent grouping of activities (see Supplementary Results and Figure S2). The right panel of Fig. 3 shows comparisons of the raw means of the global IES of dependent and non-dependent participants for each activity. The differences in global IES are significant for all WRAs and IADLs. Global IES associated with dependency only on dressing, feeding, and bathing. Executive, visual, language and memory IES showed similar trends (Figure S3).

**Fig. 4:**
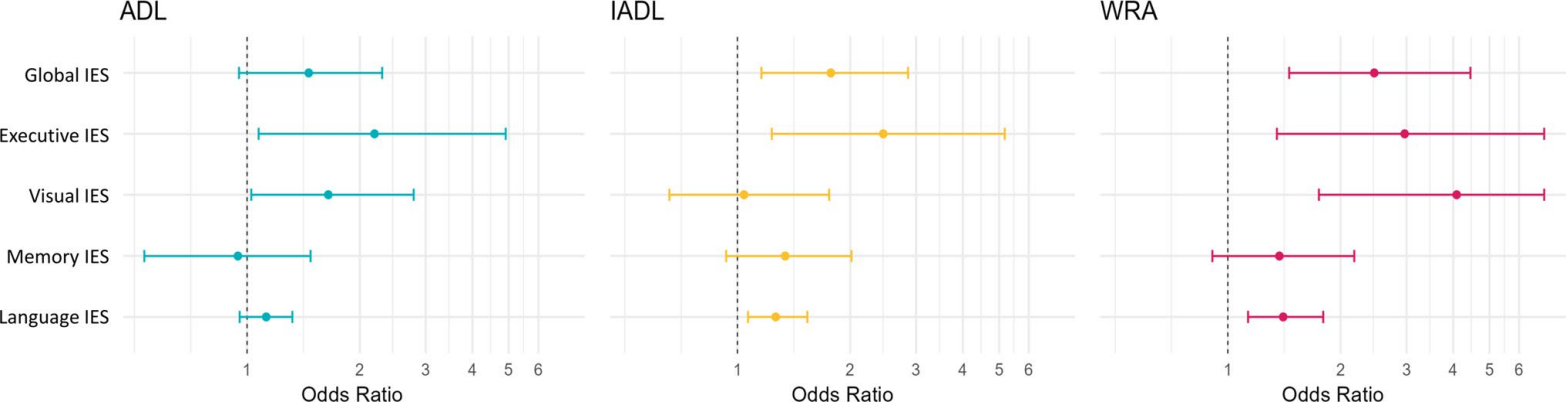
Association between cognitive performance in SelfCog IES and risk of dependence in basic and instrumental activities of daily living as well as work-related activities. ADL = activities of daily living; IADL = instrumental ADL; WRA = work-related activities; IES = inverted efficiency score. Odds Ratios from multivariate logistic models adjusted for age, number of years of study and motor IES

Logistic models revealed significant associations between SelfCog cognitive scores, except memory IES, and dependency in ADLs, IADLs and WRAs (Fig. 4). Each additional point in the global IES was associated with an increased risk of dependence in IADLs (OR[95% CI] = 1.78[1.16–2.86] and WRAs (OR[95% CI] = 2.46[1.46–4.45]). Increased executive IES was associated with increased risk of dependence in ADLs (OR[95% CI] = 2.19[1.07–4.91]), IADL (OR[95 %CI] = 2.45[1.23–5.18]), and WRAs (OR[95% CI] = 2.97[1.35–7.23]). Increased language IES was associated with increased dependence in IADLs (OR[95% CI] = 1.27[1.07–1.54]) and WRAs (OR[95% CI] = 1.41[1.13–1.80]). In addition, increased visual IES was associated with increased dependence in WRAs (OR[95% CI] = 4.09[1.75–811.03]). Motor IES was significantly associated with all groups of activities regardless of the cognitive domain included in the model. Each additional motor IES point was associated with an increased risk of dependence in ADLs by a factor of 8, in IADLs by a factor of more than 100, and in WRAs by a factor of more than 16.

The most complex IADLs (managing finances, groceries, laundry, transport, and housekeeping) were associated with low performance in all cognitive domains (*p* < 0.024 for all, Figure S4). In contrast, inability to drive was not associated with any cognitive domain but was strongly associated with motor IES (log-odds[95% CI] = 5.5[3.74–7.61]).

### Post hoc* results*

Of the participants, 153 completed the PBA-s. Participants with a TFC < 13 had higher probability to have a higher apathy, irritability and obsessive–compulsive disorders than those with a TFC = 13 (for apathy mean(SD) = 2.51(3.53) vs 0.67(2.20), respectively, *p* < 0.001; for irritability mean(SD) = 2.40(3.75) vs 1.14(2.08), respectively, *p* = 0.027; for obsessive–compulsive disorders mean(SD) = 2.48(3.84) vs 0.25(1.02), respectively, *p* < 0.001). Depression score showed no association with TFC. All participants here scored 0 at the psychosis evaluation. In fully adjusted models, apathy and obsessive–compulsive disorders were shown to be robustly associated with loss of functional capacity, and irritability associated with functional capacity principally measured with TFC (Table S4-S7). Regarding pharmacological treatment, 109 had complete data and those with a TFC < 13 compared to those with TFC = 13 showed a higher probability of being exposed to antidepressants (84.4% vs 50.0%, *p* < 0.001), and anxiolytics (65.6% vs 15.2%, *p* < 0.001). Antidepressants and antipsychotics were associated with functional limitations measured by TFC and IS independently of cognitive and motor scores mainly with SelfCog IES (Table S8-S9). All tolerance values and coefficients for cognitive and motor variables showed similar trends to the main results, irrespective of the neuropsychiatric symptom or pharmacological treatment included in the model.

In phenotype cluster analysis was identify 2 groups of participants, those with slight or absent motor symptoms with mildly variability of cognitive symptoms (*n* = 89) and those with marked motor symptoms and more variability cognitive symptoms, tending toward greater impairment (*n* = 69, Figure S5, Panel A). In the fully adjusted models including the interaction between cognitive tests and phenotypes, the coefficients and tolerances remained similar to the main analyses. Phenotypes were shown to interact significantly with global, memory, executive and language IES and their association with functional impairment. In these models we observed that cognitive performance was not significantly associated with loss of function in the group with mild motor and cognitive symptoms (Figure S5, Panel B). On the other hand, the phenotypes interacted robustly with SDMT and association with functional impairment but maintained the same trends as the main results (Figure S5, Panel C).

### Sensitivity results

Bayesian longitudinal analyses showed similar trends to the main results, but the associations of visual and language scores with TFC and IS were less strong (Figure S5). Analyses with normalized cognitive tests and TMS as an adjustment variable showed similar trends to the main analyses, but with reduced or no longer significant associations (Figure S6). The logistic regressions performed with propensity scores as weights, or without imputation of UHDRS cognitive data, showed trends similar to the main results, except that the associations with ADLs were no longer significant (see Figures S7-S8).

## Discussion

We independently assessed the impact of cognitive and motor impairments on functional limitations as measured by the TFC, IS, and FCS, in a cohort of 158 HD mutation carriers. Motor abilities were measured with SelfCog motor score and the TMS, while cognitive abilities were measured with the SelfCog and the UHDRS cognitive scores, the advantage of SelfCog over the UHDRS being its ability to measure cognitive and motor domains independently. Motor impairments showed the strongest associations with functional limitations, even after adjusting for cognitive deficits. Within the different cognitive domains assessed with the SelfCog, global cognitive and executive deficits of the SelfCog contributed more to functional decline than language, visuospatial and memory deficits. High global cognitive and executive deficits were associated with an increased probability of dependence in ADLs, IADLs, and WRAs. High visuospatial and language deficits were associated with an increased probability of dependence in WRAs, with visuospatial deficits being more associated with dependence in ADLs and language deficits with dependence in IADLs.

This study confirmed that motor skills are the major contributor to functional capacity followed by cognitive performance [45]. Although the association between motor and cognitive deficits with loss of functional capacity in HD is well documented [6, 8, 25, 45, 46], this study, using the SelfCog, provides insight into the specific contribution of each cognitive domain independently of motor impairments. In adjusted model for motor scores, compared to the UHDRS cognitive scores, the SelfCog IES demonstrated better specific predictive capacity (tolerance), indicating a more independent measure of the association. The post hoc analysis revealed a strong interaction between the SDMT and phenotype groups. In the stratified analysis, the SDMT remained significantly associated with functional decline in the phenotype group characterized by slight motor symptoms and likely no cognitive symptoms (Figure S4, panel B-C). This may be explained by the motor component of the SDMT. Conversely, when stratified using the SelfCog IES, cognitive symptoms were not significantly associated to functional decline in the same phenotype group, suggesting that the SelfCog IES might better differentiate cognitive symptoms compared to other cognitive tests. Nerveless, further studies are needed to confirm this hypothesis.

The effect of HD symptoms has so far been assessed on each activity of daily living individually, rather than considering ADLs, IADLs, and WRAs as groups of activities [7, 8, 22, 24, 47]. Here, we identified distinct associations of motor factors and cognitive domains with different activity groups. Overall, cognitive deficits have a greater impact on IADLs and WRAs dependence than on ADLs. Furthermore, our findings highlight the predominant role of executive functions as a contributing factor to dependence across ADLs, IADLs, and WRAs. Prior research has suggested that executive dysfunction may have adverse effects on work performance [48, 49]. Here, we show that executive dysfunction is a determinant factor in loss of autonomy in IADLs and relatively less complex ADLs. In addition, our findings indicate that visuospatial impairment significantly contributes to dependence in WRAs. Atrophy of the visual cortex, which is present early in HD particularly in associative areas such as lingual and fusiform gyri, and the lateral occipital cortex [50], affects complex perceptive tasks, object recognition, or reading [51, 52] and may lead to difficulties in the execution of work activities. Furthermore, we found that financial management, housekeeping, transportation, laundry, and grocery shopping, engage all cognitive domains, with the exception of the visuospatial domain [13, 23, 24]. Autonomy in IADLs, and in particular WRAs that depends on visuospatial abilities may be affected well before the observation of a general loss of functionality and dependence in ADLs [53].

One of the main public health objectives is to reduce the period of disability despite the onset of age-related morbidities, as life expectancy increases [17, 54]. One method to evaluate this objective is to assess dependence in ADLs/IADLs in individuals with chronic diseases and in the general population [15, 17]. Our findings indicate that disability may start in HD with loss of autonomy in IADLs associated with cognitive impairments in the early stages. Furthermore, with the onset of motor symptoms and dependence in ADLs, disability in HD may start earlier than in other neurodegenerative desease [55, 56]. Further research is needed to determine whether in HD, as in other diseases [55, 56], the time to disability during HD manifestation is reduced and whether it is modified by ongoing interventions.

Previous research has indicated that autonomy in activities of daily living, such as ADLs/IADLs, has an impact on health-related quality of life (HRQoL) [25, 45, 57, 58]. QoL of premanifest HD may be comparable to that of the healthy controls and a decline in QoL is observed in patients who have developed motor symptoms and lost their autonomy. This leads to the hypothesis that even in the presence of mild cognitive impairment associated with dependence in IADLs and WRAs, potential compensatory systems are maintaining QoL (e.g., GPS, alarms, task modifications) as observed in other conditions [59]. However, the loss of autonomy in ADLs resulting from the combination of motor and cognitive symptoms has a significant negative impact on QoL [58]. Therefore, interventions aimed at managing motor and cognitive symptoms to prevent loss of autonomy and promoting compensatory modalities (assistive devices, activity modification) may be effective strategies for maintaining HRQoL in individuals with HD.

Altogether our results highlight the specific contribution of cognitive and motor symptoms on various functional capacities and may guide future intervention. Our study was based on cross-sectional data, which might limit its generalizability. Nevertheless, sensitivity analyses with a subsample of longitudinal data and analysis with the addition of a propensity score, allowed us to observe similar trends as in the main analysis, reducing the potential for selection or methodological biases. Another limitation is that a sex effect and presumably a cultural effect were observed for some items [45, 60]. Some male participants were able to perform difficult items such as driving or finances, but not food preparation or watching children suggesting that item selection may be gender biased. However, in this study, no significant interaction of sex with cognitive assessment on functional limitations was observed in linear or logistic regression. Neuropsychiatric symptoms, and potentially their pharmacological treatment, may be associated with functional decline and represent a confounding factor that should be considered. However, given that psychiatric symptoms, unlike cognitive and motor symptoms, may be intermittent during all stages of the disease and do not follow an evolution aligned with the HD progression [38], we lack the statistical power, population diversity, and longitudinal data to include psychiatric symptoms in the main analyses. This complex challenge was also reported in previous studies [38, 41, 61, 62]. Nevertheless, a post-hoc analysis with PBA-s scores as well as pharmacological treatments was included in the fully adjusted models, and the contribution of motor and cognitive deficits remained similar to that presented in the main summaries. However, further research may be required to identify the specific impact of psychiatric symptoms on functional decline. Finally, in our cohort, one-third of HD participants did not have any functional limitation in ADLs; in fact, the BioHD and RepairHD studies were composed principally of participants with early-stage HD and mild functional limitations. Indeed, participants with severe functional limitations may have more difficulty completing the cognitive tests, resulting in under-representation of this HD population with advanced deficits and a possible underestimation of associations. However, a sensitivity analysis with propensity scores giving more weight to lower cognitive performance as would be observed in advanced HD stages [63, 64] showed similar results of associations as in the main results. Although the results of the main and sensitivity analyses are consistent, the possibility of selection bias cannot be discarded. Our findings should be interpreted considering that we focused on a population of individuals who are currently the target of major therapeutic efforts [65]. These patients, predominantly in HD ISS stages 0–2 [36, 65], are of particular interest in clinical trials, as they represent a group where interventions aim to improve symptoms and prevent the partial or total loss of autonomy or QoL. Further studies should be performed to assess the specific impact of a major cognitive deficit on the functioning of HD patients in a larger population.

## Conclusion

This study suggests that motor and cognitive deficits are independently associated with loss of functional capacity. Motor deficits showed the strongest association, followed by executive deficits and global cognitive impairments. Poor performance in executive functions exhibited a robust association with dependence in ADLs, IADLs and WRAs, suggesting that it is a marker of loss of autonomy in early HD and may guide the development of targeted interventions to preserve patients’ autonomy and quality of life.

## Supplementary Information

Below is the link to the electronic supplementary material.

Supplementary file1 (DOCX 1702 KB)

## Data Availability

The datasets generated during and/or analyzed during the current study are not publicly available due to sensitive patient information but are available from the corresponding author on reasonable request.
