# Supplementary material for: Specific contribution of cognitive and motor impairments with functional capacity and dependence in Huntington’s disease
Source: J Neurol. 2025 Feb 22;272(3):224. doi: 10.1007/s00415-025-12982-9 (PMC11846732; doi:10.1007/s00415-025-12982-9)
Supplement: Supplementary file 1 — Supplementary file1 (DOCX 1702 KB) [file 415_2025_12982_MOESM1_ESM.docx]

**Supplemental material**

***Supplementary methods***

***Imputation method***

Out of the 173 participants who completed the SelfCog assessment, 14 were missing data on the SDMT, Stroop colour/word/interference, or Literal fluency tests. To address this issue, we used an iterative imputation method based on random forest (missForest). This method enables the simultaneous imputation of missing data using the built-in out-of-bag error estimates of random forest. The comparison between the imputed dataset and the source dataset was conducted until the prediction error was minimized (normalized root mean square error). Calibration of the random forest was performed on 504 participants after excluding controls and HD participants with missing data from the TFC, IS, or FCS. Predictor variables included UHDRS cognitive tests, age, CAG repeats, years of education, sex, Selfcog IES, and total scores for TFC, IS, FCS and TMS.

***Selection and evaluation of internal consistency of basic and instrumental activities of daily living (ADL/IADL) and work-related activities (WRA).***

We first assessed the structure of the FCS items using exploratory factor analysis performed iteratively, excluding items that did not significantly contribute to any factor, showed multicollinearity, or were not considered an ADL or IADL by prior literature^58,59^. The grouping of items into factors was estimated using the minimum residual (MR) method and a tetrachoric correlation matrix. This ensured that we were able to assess the limitations in the different groups of activities separately. Second, we evaluated the internal consistency of the activities grouped into factors and the concordance between the difficulty in performing item-related tasks and the phenotype of HD individuals using RASCH analysis. The model was built using conditional maximum likelihood for parameter estimation, and items with infit t-statistics greater than 2 were considered to distort the model. The correspondence between the resulting factors, their respective items, and ADLs, IADLs, and WRAs was then carried out.

***Supplementary results***

***Selection of basic and instrumental activities of daily living (ADL/IADL) and work-related activities (WRA).***

The selection of activities of daily living and their regrouping into a consistent structure carried out with the exploratory factor and Rasch analyses of the FCS items allows for the discernment of 7 ADLs (Bathing, Dressing, Feeding, Grooming, Mobility, Toileting and Transfer), 10 IADLs (Housekeeping, Finances, Food preparation, Grocery shopping, Medications, Purchases, Driving, Telephone use, Laundry, and Transportation), and 3 WRA (Regular work, Modified work, Volunteering). The analysis grouped the items into four factors: the first and fourth factor regroups the WRAs and IADLs, while the second and third factors regroup the ADLs (Figure S2). These groupings establish the items necessary to determine whether an individual is dependent on each type of activity separately. Collinear items were merged, thus, items “*walking”* and “*walking with assistance”* were grouped into “*mobility”,* and “*transferring from a chair”* and “*getting out of bed”* were grouped into “*transfer”.* The item “*Home care*” was omitted due to the high correlation with the remaining FCS items (Correlation≥0.76). The item about “*moving around the neighbourhood*” was excluded because it is not significantly associated with any factor. Finally, the item concerning *child supervision* was excluded as it distorted the RASCH model (infit t-statistic = 2.05). The items "*food preparation*" presented infit t-statistics slightly above 2 but were retained due to their relevance in the literature of daily activities.

The Rasch model selected from the studied population identified ADLs as relatively easy to perform compared to IADLs and WRAs (Figure 3, left panel). Among the ADLs, dressing was the most challenging activity (logit=-2.11), while toileting and transfers were the easiest (logit=-3.41 for both). The IADLs with the lowest level of difficulty were telephone use (logits=-2.61) while driving was the most difficult of IADLs (logits=3.34). Regular and modified work were the items more challenging (logits=7.75 and 4.40 respectively) compared to the rest of the items.

**Figure S1. Association of normalized cognitive capacity scores with the UHDRS functional limitation scales.**

**
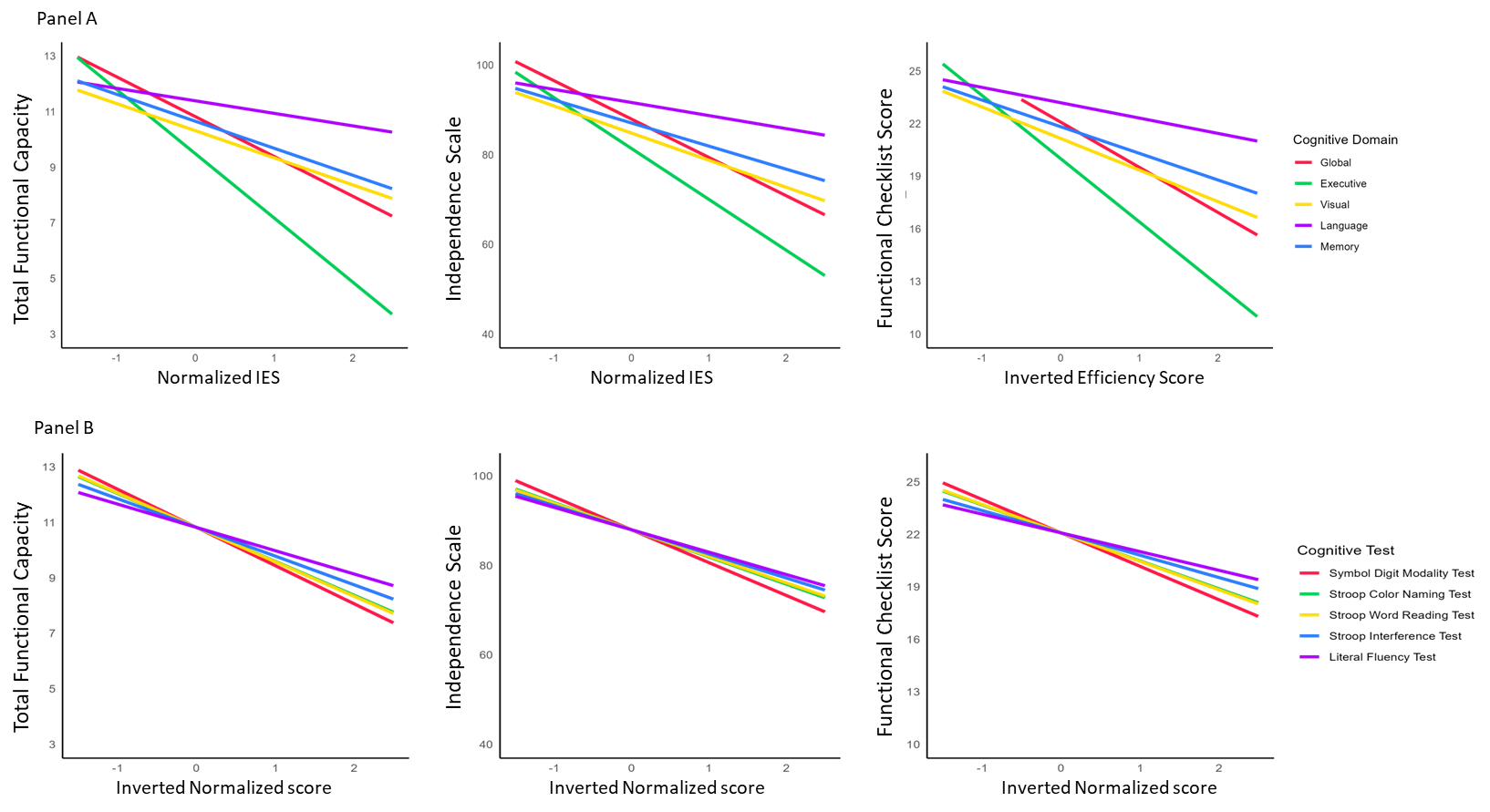
**

Panel A: Association between the Selfcog global and domain IES and functional limitations scales. Scoring was normalized using the overall mean and standard deviation of all scores together by the measure of functional limitation. Panel B: Association between the UHDRS cognitive test and functional limitations scales. Scoring was normalized using the mean and standard deviation of each score by the measure of functional limitation. The normalized scores were inverted (multiplied by -1) for comparison with the IES scores. Predictions of multivariate linear models, adjusted for age, number of years of study and motor IES (Table S1). Normalized coefficients in Table S-model 3. Graphic with Raw Score in Figure 2.

**Figure S2. Diagram of the exploratory factor analysis of the functional Checklist Score items.**

**
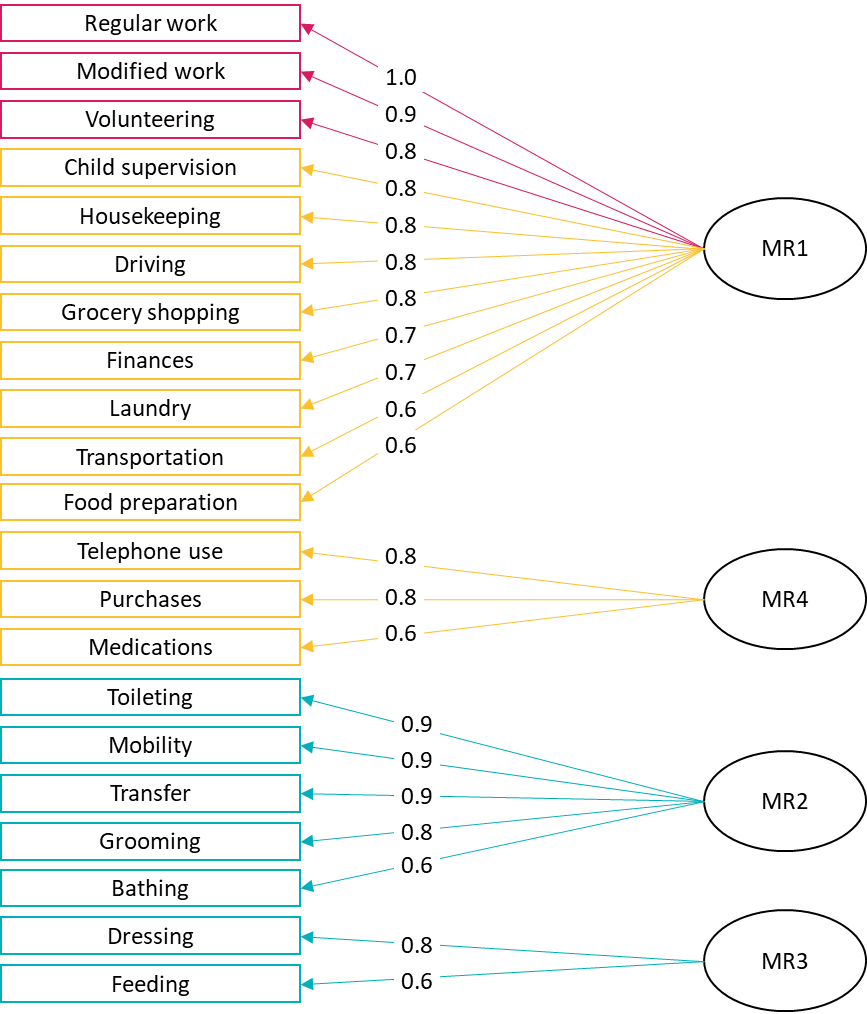
**

**
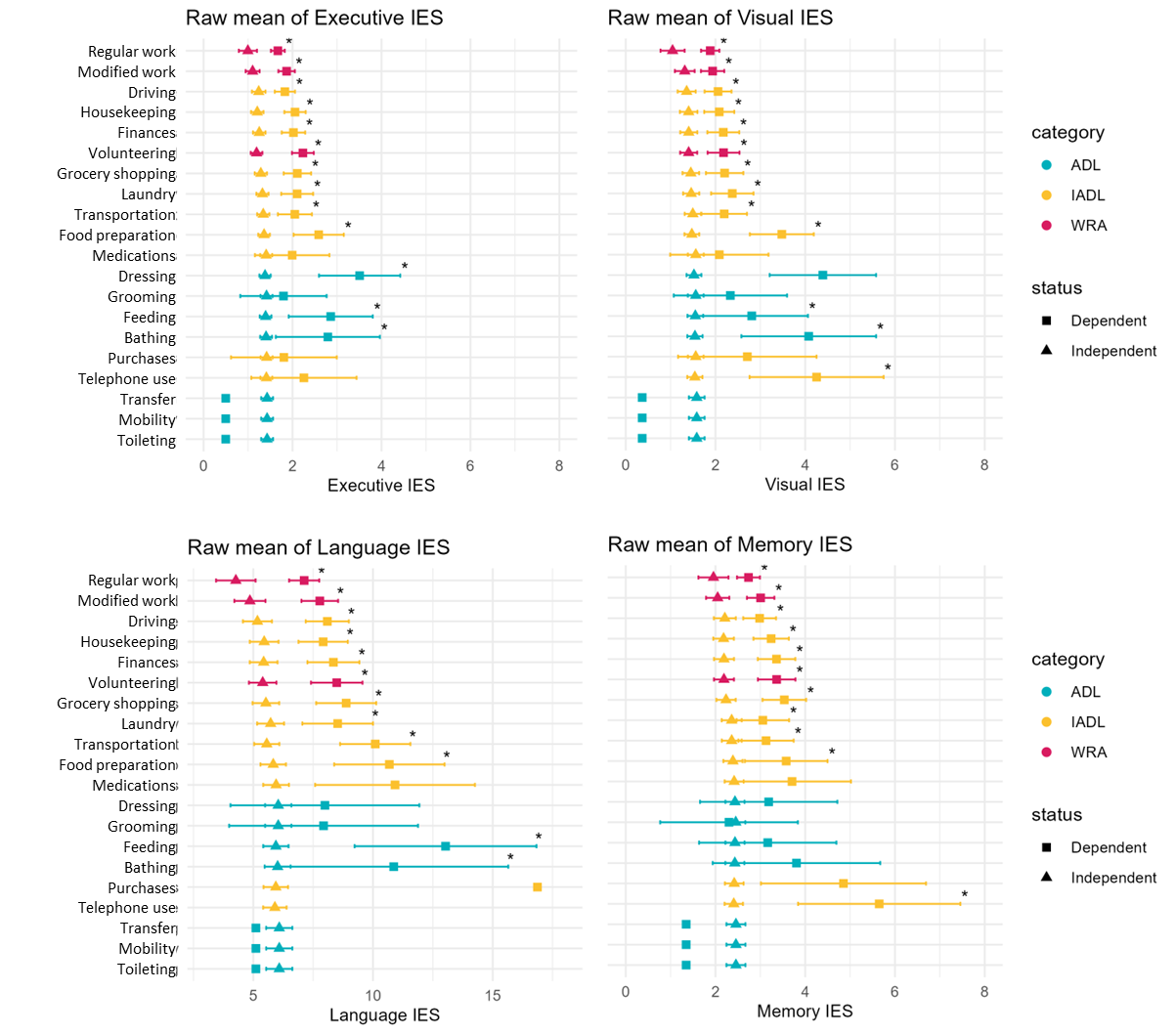
Figure S3. Raw mean comparison between dependent and independent participants for each ADL, IADL and WRA.**

*Raw mean and confidence interval at 95%. ADL= activities of daily living; IADL= instrumental ADL; WRA= work-related activities, *=significant mean difference (p<0.05).*

**Figure S4. Association between cognitive performance in SelfCog IES and the risk of dependence in specific activities.**

**
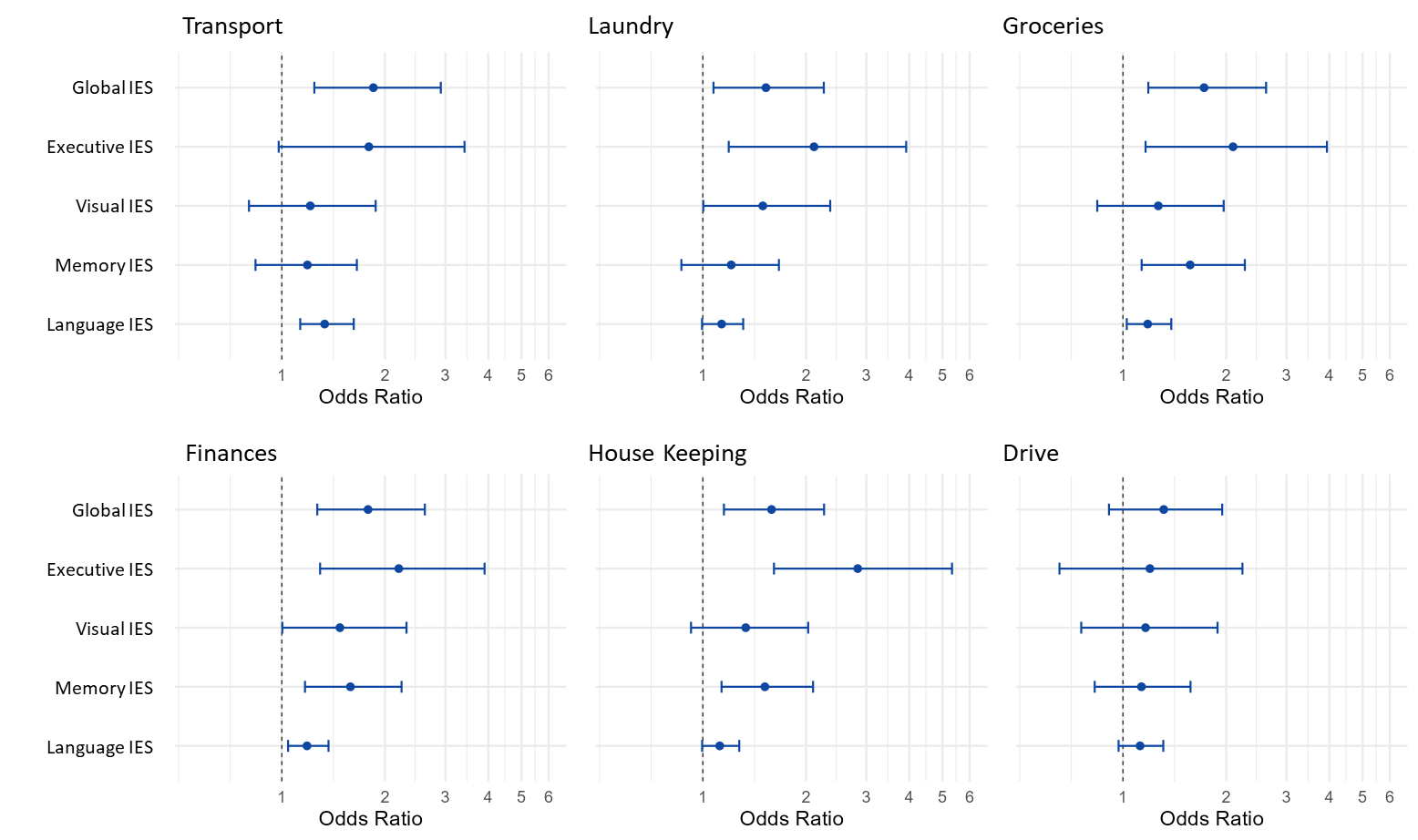
**

*Odds Ratios from multivariate logistic models adjusted for age, number of years of study and motor IES.*

***
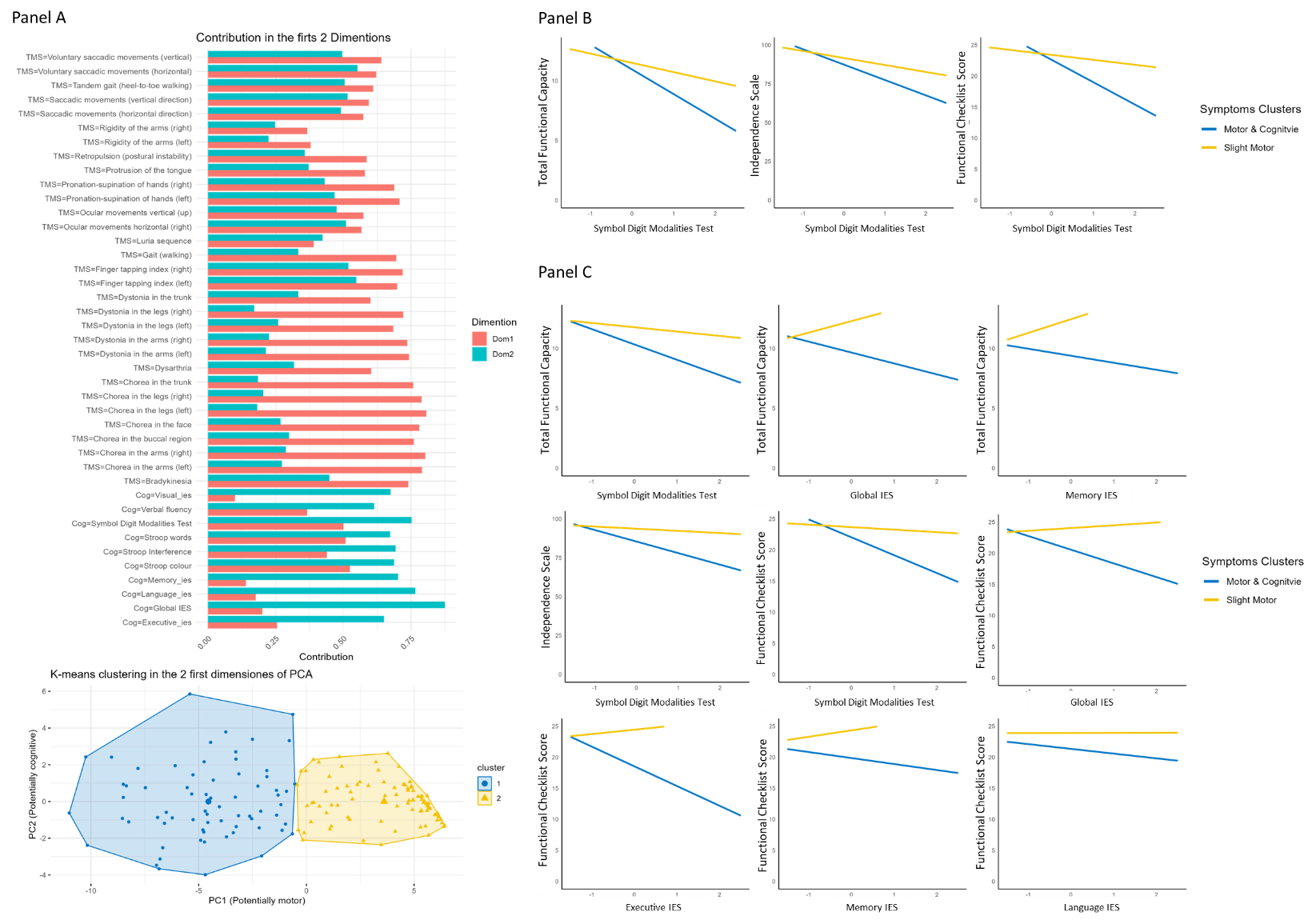
Figure S5. Association of normalized cognitive capacity scores with the UHDRS functional limitation scales stratified by Symptoms Clusters*.***

**All participants were included in the cluster analysis. Motor variables were extracted from the specific items of the Total Motor score (TMS) and all cognitive tests evaluated in this study were included. Principal component analysis (PCA) and a k-means method was used to identify two different symptom phenotypes: 1=motor and cognitive symptoms and 2= slight motor symptoms. Panel A: PCA summary and cluster analysis. Panel B: Association between cognitive test and functional limitations stratified by symptom phenotype. Panel C: Association between cognitive test and functional limitations stratified by symptomatic phenotype. Only models with significant interaction between cognitive tests and phenotype are presented.*

***Figure S6. Association of cognitive domains and Functional Limitations in ADL, IADL and Work activities in longitudinal data with Bayesian approach***

**
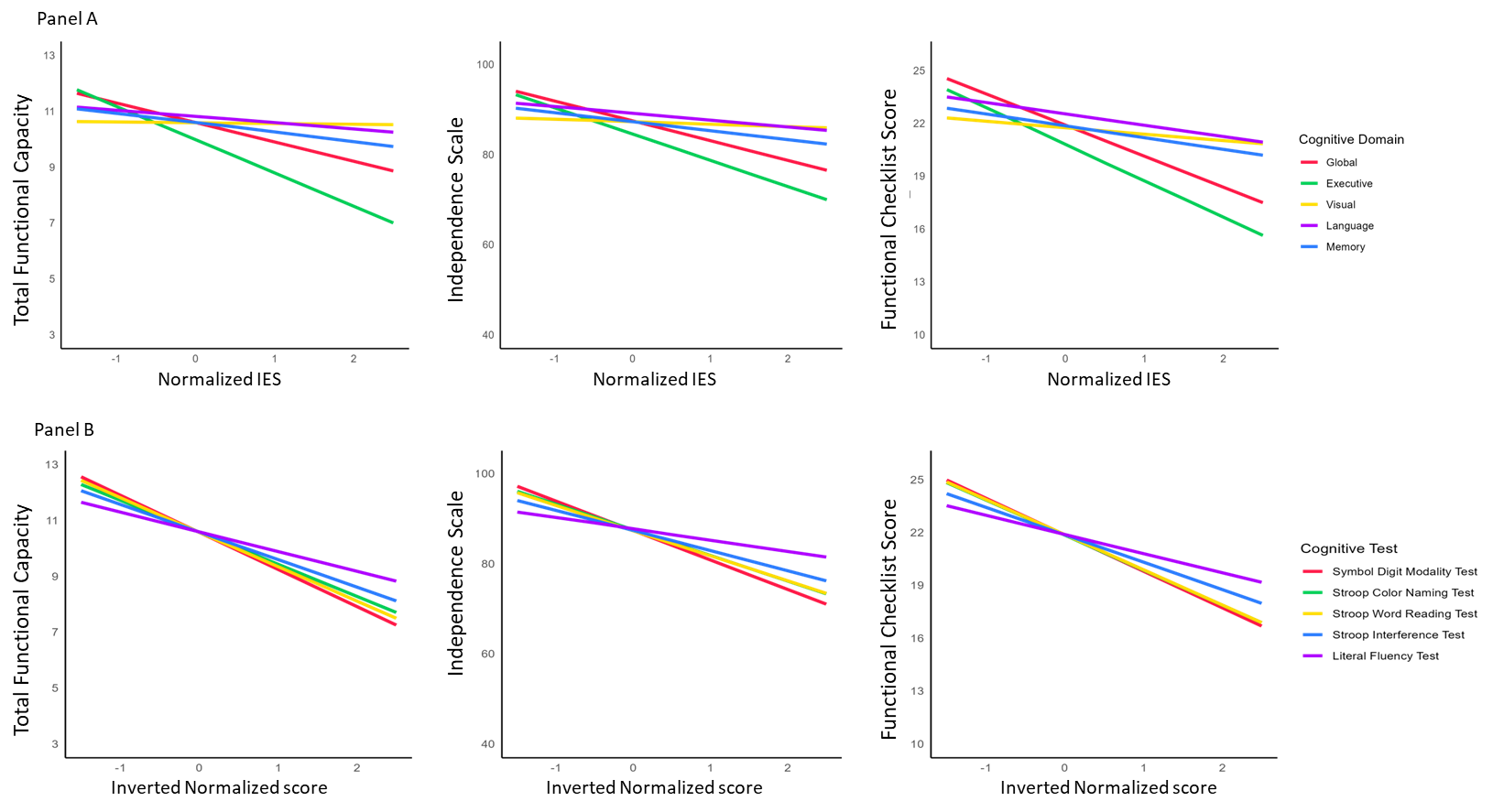
**

*Panel A: Association between the Selfcog global and domain IES and functional limitations scales. Scoring was normalized using the overall mean and standard deviation of all scores together by the measure of functional limitation. Panel B: Association between the UHDRS cognitive test and functional limitations scales. Scoring was normalized using the mean and standard deviation of each score by the measure of functional limitation. The normalized scores were inverted (multiplied by -1) for comparison with the IES scores. Predictions of multivariate linear mixed models with random effect at individual level and intercept and slope. Model adjusted for age, number of years of study and motor IES. For the Bayesian approach with metropolis Hasting’s algorithm: 4 chains, 4000 iterations, 1000 for warmup.*

**Figure S7. Association of cognitive domains and Functional Limitations in ADL, IADL and Work activities adjusting for Total Motor Score.**

**
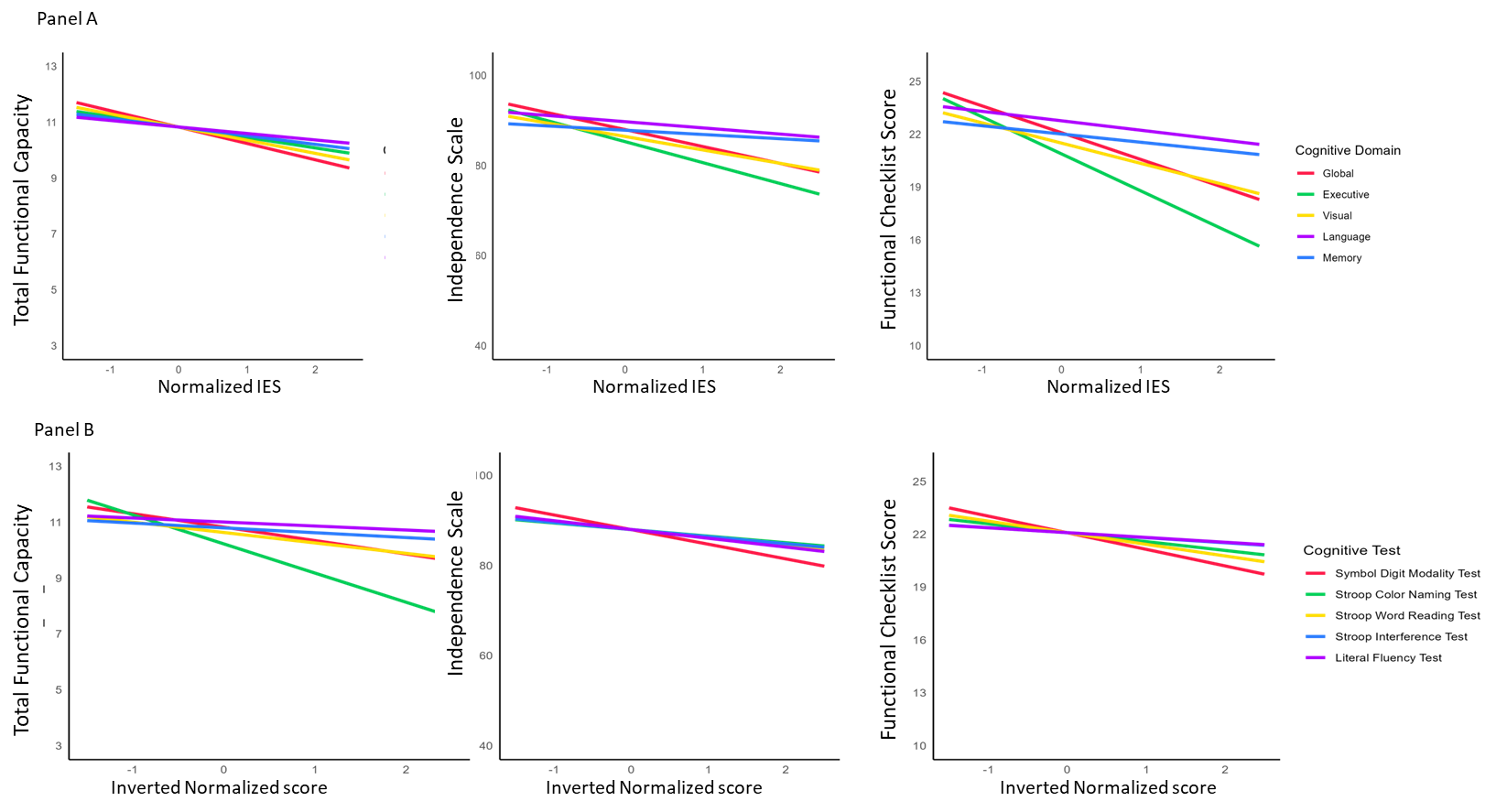
**

*Panel A: Association between the Selfcog global and domain IES and functional limitations scales. Scoring was normalized using the overall mean and standard deviation of all scores together by the measure of functional limitation. Panel B: Association between the UHDRS cognitive test and functional limitations scales. Scoring was normalized using the mean and standard deviation of each score by the measure of functional limitation. The normalized scores were inverted (multiplied by -1) for comparison with the IES scores. Predictions of multivariate linear models, adjusted for age, number of years of study and total motor Score. Table S2-model 3.*

**Figure S8. Association between cognitive performance in SelfCog IES and the risk of dependence in basic and instrumental activities of daily living as well as work-related activities (model with propensity scores)**

**
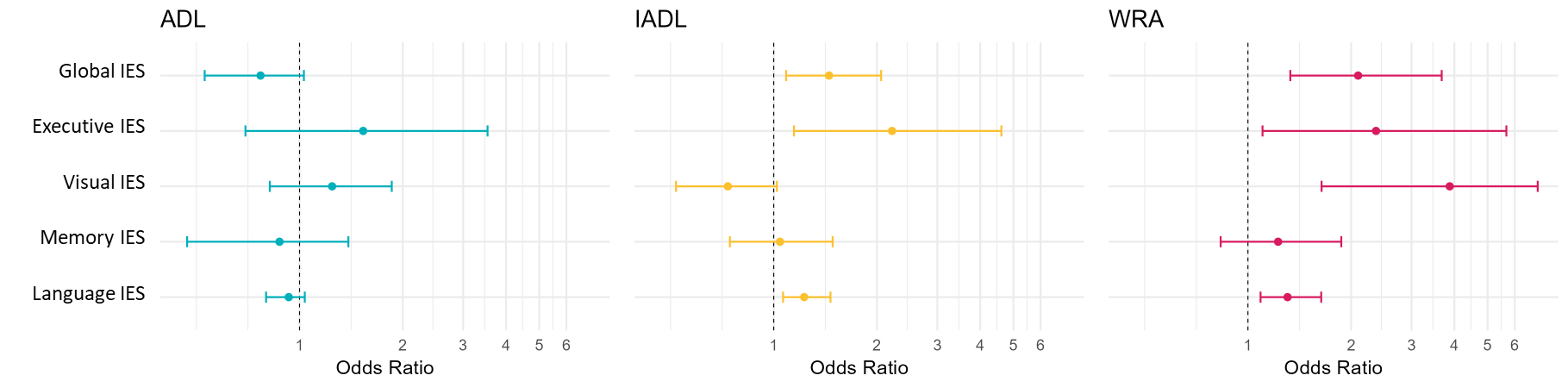
**

*ADL= activities of daily living; IADL= instrumental ADL; WRA= work-related activities; IES= inverted efficiency score. Odds Ratios from multivariate logistic models adjusted for age, number of years of study and motor IES.*

**Figure S9. Association of cognitive capacity with the measure of functional limitation (no imputed data)**

**
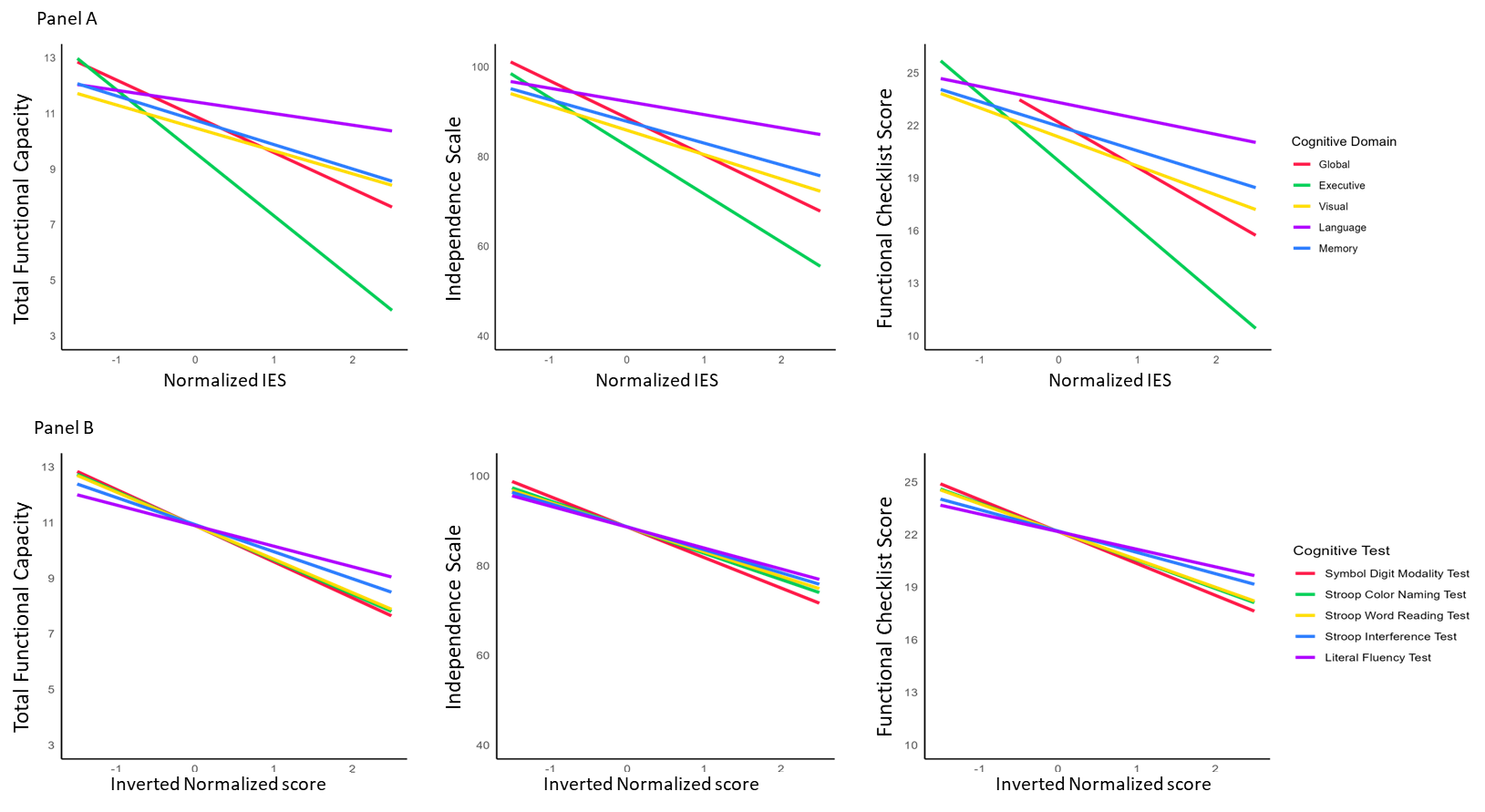
**

*Panel A: Association between the Selfcog global and domain IES and functional limitations scales. Scoring was normalized using the overall mean and standard deviation of all scores together by the measure of functional limitation. Panel B: Association between the UHDRS cognitive test and functional limitations scales. Scoring was normalized using the mean and standard deviation of each score by the measure of functional limitation. The normalized scores were inverted (multiplied by -1) for comparison with the IES scores. Predictions of multivariate linear models, adjusted for age, number of years of study and motor IES. Model calibration with data from 144 individuals.*

|  | **Type 1 models** | |  | **Type 2 models** | | | |  | **Type 3 models** | | | |
| --- | --- | --- | --- | --- | --- | --- | --- | --- | --- | --- | --- | --- |
|  |  |  |  | **Cognitive Score** | | **Motor IES** | |  | **Cognitive Score** | | **Motor IES** | |
|  | **Coefficient (95%IC)** | **p-value** |  | **Coefficient (95%IC)** | **p-value** | **Coefficient (95%IC)** | **p-value** |  | **Coefficient (95%IC)** | **p-value** | **Coefficient (95%IC)** | **p-value** |
| **Total Functional Score** | |  |  |  |  |  |  |  |  |  |  |  |
| Global IES | -0.86 (-1.07 to -0.64) | <0.001 |  | -0.60 (-0.85 to -0.36) | <0.001 | -2.06 (-3.12 to -0.99) | <0.001 |  | -0.57 (-0.81 to -0.32) | <0.001 | -1.82 (-2.91 to -0.73) | 0.001 |
| Executive IES | -1.36 (-1.72 to -1.00) | <0.001 |  | -0.95 (-1.34 to -0.56) | 0.006 | -2.24 (-3.27 to -1.21) | <0.001 |  | -0.92 (-1.31 to -0.52) | <0.001 | -2.04 (-3.08 to -0.99) | <0.001 |
| Visual IES | -0.77 (-1.07 to -0.47) | <0.001 |  | -0.42 (-0.72 to -0.12) | <0.001 | -2.85 (-3.89 to -1.81) | <0.001 |  | -0.39 (-0.69 to -0.09) | 0.012 | -2.51 (-3.58 to -1.44) | <0.001 |
| Language IES | -0.30 (-0.39 to -0.21) | <0.001 |  | -0.19 (-0.29 to -0.09) | <0.001 | -2.49 (-3.54 to -1.44) | <0.001 |  | -0.18 (-0.27 to -0.08) | <0.001 | -2.14 (-3.22 to -1.05) | <0.001 |
| Memory IES | -0.67 (-0.91 to -0.42) | <0.001 |  | -0.43 (-0.67 to -0.19) | 0.001 | -2.84 (-3.83 to -1.84) | <0.001 |  | -0.38 (-0.62 to -0.15) | 0.002 | -2.53 (-3.56 to -1.49) | <0.001 |
| SDMT | 0.10 (0.09 to 0.12) | <0.001 |  | 0.09 (0.07 to 0.12) | <0.001 | -0.70 (-1.72 to 0.32) | 0.176 |  | 0.09 (0.07 to 0.11) | <0.001 | -0.59 (-1.62 to 0.44) | 0.257 |
| Stroop colour | 0.08 (0.07 to 0.09) | <0.001 |  | 0.07 (0.05 to 0.09) | <0.001 | -0.97 (-2.04 to 0.10) | 0.074 |  | 0.07 (0.05 to 0.09) | <0.001 | -0.88 (-1.96 to 0.2) | 0.108 |
| Stroop words | 0.06 (0.05 to 0.07) | <0.001 |  | 0.05 (0.04 to 0.07) | <0.001 | -0.95 (-2.00 to 0.09) | 0.073 |  | 0.05 (0.04 to 0.07) | <0.001 | -0.77 (-1.84 to 0.3) | 0.159 |
| Stroop Interference | 0.11 (0.09 to 0.13) | <0.001 |  | 0.09 (0.06 to 0.12) | <0.001 | -1.55 (-2.58 to -0.52) | 0.003 |  | 0.08 (0.06 to 0.11) | <0.001 | -1.51 (-2.56 to -0.46) | 0.005 |
| Verbal fluency | 0.09 (0.07 to 0.12) | <0.001 |  | 0.07 (0.04 to 0.09) | <0.001 | -2.23 (-3.23 to -1.24) | <0.001 |  | 0.07 (0.04 to 0.09) | <0.001 | -1.75 (-2.81 to -0.69) | 0.001 |
| *Motor IES* | *-3.46 (-1.45 to -0.67)* | *<0.001* |  |  |  |  |  |  |  |  |  |  |
| **Independence scale** |  |  |  |  |  |  |  |  |  |  |  |  |
| Global IES | -4.94 (-6.00 to -3.88) | <0.001 |  | -3.58 (-4.77 to -2.39) | <0.001 | -11.04 (-16.22 to -5.86) | <0.001 |  | -3.39 (-4.57 to -2.21) | <0.001 | -9.76 (-15.01 to -4.51) | <0.001 |
| Executive IES | -7.19 (-9.04 to -5.35) | <0.001 |  | -4.76 (-6.72 to -2.80) | 0.001 | -13.29 (-18.47 to -8.12) | <0.001 |  | -4.5 (-6.46 to -2.53) | <0.001 | -12.06 (-17.29 to -6.83) | <0.001 |
| Visual IES | -4.50 (-6.00 to -3.00) | <0.001 |  | -2.59 (-4.09 to -1.10) | <0.001 | -15.62 (-20.78 to -10.47) | <0.001 |  | -2.4 (-3.87 to -0.92) | 0.002 | -13.76 (-19.04 to -8.48) | <0.001 |
| Language IES | -1.80 (-2.25 to -1.34) | <0.001 |  | -1.21 (-1.69 to -0.74) | <0.001 | -13.17 (-18.29 to -8.05) | <0.001 |  | -1.15 (-1.62 to -0.69) | <0.001 | -11.23 (-16.48 to -5.98) | <0.001 |
| Memory IES | -3.63 (-4.88 to -2.38) | <0.001 |  | -2.28 (-3.47 to -1.09) | <0.001 | -16.05 (-21.00 to -11.09) | <0.001 |  | -2.04 (-3.21 to -0.87) | 0.001 | -14.29 (-19.42 to -9.15) | <0.001 |
| SDMT | 0.57 (0.49 to 0.65) | <0.001 |  | 0.51 (0.40 to 0.61) | <0.001 | -4.62 (-9.57 to 0.32) | 0.067 |  | 0.49 (0.38 to 0.59) | <0.001 | -3.95 (-8.91 to 1.00) | 0.117 |
| Stroop colour | 0.42 (0.35 to 0.50) | <0.001 |  | 0.35 (0.25 to 0.44) | <0.001 | -6.88 (-12.24 to -1.53) | 0.012 |  | 0.33 (0.24 to 0.42) | <0.001 | -6.26 (-11.63 to -0.89) | 0.023 |
| Stroop words | 0.32 (0.26 to 0.38) | <0.001 |  | 0.26 (0.19 to 0.33) | <0.001 | -7.26 (-12.57 to -1.96) | 0.008 |  | 0.25 (0.17 to 0.32) | <0.001 | -6.11 (-11.52 to -0.7) | 0.027 |
| Stroop Interference | 0.60 (0.49 to 0.72) | <0.001 |  | 0.47 (0.34 to 0.60) | <0.001 | -9.35 (-14.43 to -4.26) | <0.001 |  | 0.43 (0.3 to 0.57) | <0.001 | -9.02 (-14.18 to -3.86) | 0.001 |
| Verbal fluency | 0.54 (0.42 to 0.65) | <0.001 |  | 0.40 (0.28 to 0.52) | <0.001 | -12.15 (-16.99 to -7.32) | <0.001 |  | 0.39 (0.27 to 0.51) | <0.001 | -9.35 (-14.45 to -4.25) | <0.001 |
| *Motor IES* | *-19.38 (-1.45 to -0.67)* | *<0.001* |  |  |  |  |  |  |  |  |  |  |
| **Functional Checklist Score** | |  |  |  |  |  |  |  |  |  |  |  |
| Global IES | -1.47 (-1.80 to -1.15) | <0.001 |  | -1.03 (-1.4 to -0.66) | <0.001 | -3.62 (-5.22 to -2.02) | <0.001 |  | -1.02 (-1.39 to -0.65) | <0.001 | -3.59 (-5.22 to -1.95) | <0.001 |
| Executive IES | -2.17 (-2.73 to -1.6) | <0.001 |  | -1.39 (-1.99 to -0.79) | 0.002 | -4.24 (-5.82 to -2.65) | <0.001 |  | -1.43 (-2.04 to -0.82) | <0.001 | -4.21 (-5.82 to -2.59) | <0.001 |
| Visual IES | -1.33 (-1.79 to -0.86) | <0.001 |  | -0.72 (-1.18 to -0.26) | <0.001 | -4.97 (-6.55 to -3.39) | <0.001 |  | -0.71 (-1.17 to -0.26) | 0.002 | -4.8 (-6.44 to -3.16) | <0.001 |
| Language IES | -0.54 (-0.68 to -0.40) | <0.001 |  | -0.35 (-0.5 to -0.21) | <0.001 | -4.2 (-5.77 to -2.63) | <0.001 |  | -0.35 (-0.49 to -0.2) | <0.001 | -4.04 (-5.67 to -2.4) | <0.001 |
| Memory IES | -1.06 (-1.45 to -0.67) | <0.001 |  | -0.63 (-1 to -0.27) | 0.001 | -5.09 (-6.61 to -3.57) | <0.001 |  | -0.6 (-0.97 to -0.24) | 0.001 | -4.97 (-6.56 to -3.37) | <0.001 |
| SDMT | 0.16 (0.13 to 0.19) | <0.001 |  | 0.13 (0.09 to 0.16) | <0.001 | -2.29 (-3.93 to -0.66) | 0.006 |  | 0.13 (0.09 to 0.16) | <0.001 | -2.36 (-4.02 to -0.71) | 0.005 |
| Stroop colour | 0.12 (0.09 to 0.14) | <0.001 |  | 0.09 (0.06 to 0.12) | <0.001 | -2.87 (-4.58 to -1.15) | 0.001 |  | 0.09 (0.06 to 0.12) | <0.001 | -2.94 (-4.67 to -1.2) | 0.001 |
| Stroop words | 0.09 (0.07 to 0.11) | <0.001 |  | 0.07 (0.05 to 0.09) | <0.001 | -2.76 (-4.44 to -1.08) | 0.001 |  | 0.07 (0.04 to 0.09) | <0.001 | -2.78 (-4.51 to -1.05) | 0.002 |
| Stroop Interference | 0.16 (0.12 to 0.20) | <0.001 |  | 0.11 (0.06 to 0.15) | <0.001 | -3.74 (-5.39 to -2.08) | <0.001 |  | 0.1 (0.06 to 0.15) | <0.001 | -3.86 (-5.55 to -2.18) | <0.001 |
| Verbal fluency | 0.14 (0.10 to 0.18) | <0.001 |  | 0.09 (0.05 to 0.13) | <0.001 | -4.39 (-5.96 to -2.83) | <0.001 |  | 0.08 (0.04 to 0.12) | <0.001 | -4.11 (-5.79 to -2.42) | <0.001 |
| *Motor IES* | *-6.01 (-1.45 to -0.67)* | *<0.001* |  |  |  |  |  |  |  |  |  |  |

**Table S1. Association of raw scores of cognitive test and motor IES with and functional measures.**

*Type 1 models: Results of linear univariate models. Type 2 models: Results of linear models adjusted for Motor IES. Type 3 models: Results of linear models adjusted for Motor IES, age at visit and years of education.*

|  | **Type 1 models** | |  | **Type 2 models** | | | |  | **Type 3 models** | | | |
| --- | --- | --- | --- | --- | --- | --- | --- | --- | --- | --- | --- | --- |
|  |  |  |  | **Cognitive Score** | | **Total Motor Score** | |  | **Cognitive Score** | | **Total Motor Score** | |
|  | **Coefficient (95%IC)** | **p-value** |  | **Coefficient (95%IC)** | **p-value** | **Coefficient (95%IC)** | **p-value** |  | **Coefficient (95%IC)** | **p-value** | **Coefficient (95%IC)** | **p-value** |
| **Total Functional Score** | |  |  |  |  |  |  |  |  |  |  |  |
| Global IES | -0.86 (-1.07 to -0.64) | <0.001 |  | -0.21 (-0.43 to 0.00) | 0.048 | -0.08 (-0.09 to -0.06) | <0.001 |  | -0.19 (-0.40 to 0.02) | 0.074 | -0.08 (-0.09 to -0.06) | <0.001 |
| Executive IES | -1.36 (-1.72 to -1.00) | <0.001 |  | -0.43 (-0.76 to -0.10) | 0.01 | -0.08 (-0.09 to -0.06) | <0.001 |  | -0.41 (-0.74 to -0.09) | 0.014 | -0.08 (-0.09 to -0.06) | <0.001 |
| Visual IES | -0.77 (-1.07 to -0.47) | <0.001 |  | -0.17 (-0.40 to 0.07) | 0.171 | -0.08 (-0.10 to -0.07) | <0.001 |  | -0.15 (-0.38 to 0.09) | 0.213 | -0.08 (-0.09 to -0.07) | <0.001 |
| Language IES | -0.30 (-0.39 to -0.21) | <0.001 |  | -0.06 (-0.14 to 0.02) | 0.127 | -0.08 (-0.10 to -0.07) | <0.001 |  | -0.06 (-0.14 to 0.02) | 0.158 | -0.08 (-0.09 to -0.06) | <0.001 |
| Memory IES | -0.67 (-0.91 to -0.42) | <0.001 |  | -0.10 (-0.30 to 0.10) | 0.344 | -0.08 (-0.10 to -0.07) | <0.001 |  | -0.07 (-0.27 to 0.13) | 0.495 | -0.08 (-0.10 to -0.07) | <0.001 |
| SDMT | 0.10 (0.09 to 0.12) | <0.001 |  | 0.04 (0.02 to 0.07) | 0.001 | -0.06 (-0.08 to -0.04) | <0.001 |  | 0.04 (0.01 to 0.06) | 0.002 | -0.06 (-0.08 to -0.04) | <0.001 |
| Stroop colour | 0.08 (0.07 to 0.09) | <0.001 |  | 0.02 (0.00 to 0.04) | 0.021 | -0.07 (-0.09 to -0.05) | <0.001 |  | 0.02 (0.00 to 0.04) | 0.049 | -0.07 (-0.09 to -0.05) | <0.001 |
| Stroop words | 0.06 (0.05 to 0.07) | <0.001 |  | 0.02 (0.01 to 0.04) | 0.002 | -0.07 (-0.08 to -0.05) | <0.001 |  | 0.02 (0.01 to 0.03) | 0.008 | -0.07 (-0.08 to -0.05) | <0.001 |
| Stroop Interference | 0.11 (0.09 to 0.13) | <0.001 |  | 0.03 (0.01 to 0.06) | 0.017 | -0.07 (-0.09 to -0.06) | <0.001 |  | 0.02 (0.00 to 0.05) | 0.073 | -0.07 (-0.09 to -0.06) | <0.001 |
| Verbal fluency | 0.09 (0.07 to 0.12) | <0.001 |  | 0.02 (0.00 to 0.05) | 0.044 | -0.08 (-0.09 to -0.06) | <0.001 |  | 0.02 (-0.01 to 0.04) | 0.125 | -0.08 (-0.09 to -0.06) | <0.001 |
| *Total Motor Score* | *-0.09 (0.10 to 0.18)* | *<0.001* |  |  |  |  |  |  |  |  |  |  |
| **Independence scale** | |  |  |  |  |  |  |  |  |  |  |  |
| Global IES | -4.94 (-6.00 to -3.88) | <0.001 |  | -1.63 (-2.63 to -0.63) | 0.002 | -0.41 (-0.48 to -0.33) | <0.001 |  | -1.49 (-2.47 to -0.52) | 0.003 | -0.40 (-0.47 to -0.32) | <0.001 |
| Executive IES | -7.19 (-9.04 to -5.35) | <0.001 |  | -2.02 (-3.59 to -0.46) | 0.012 | -0.43 (-0.50 to -0.36) | <0.001 |  | -1.85 (-3.41 to -0.29) | 0.021 | -0.42 (-0.49 to -0.35) | <0.001 |
| Visual IES | -4.50 (-6.00 to -3.00) | <0.001 |  | -1.29 (-2.42 to -0.17) | 0.025 | -0.45 (-0.51 to -0.38) | <0.001 |  | -1.18 (-2.28 to -0.08) | 0.035 | -0.43 (-0.49 to -0.37) | <0.001 |
| Language IES | -1.80 (-2.25 to -1.34) | <0.001 |  | -0.58 (-0.96 to -0.20) | 0.003 | -0.42 (-0.49 to -0.35) | <0.001 |  | -0.55 (-0.91 to -0.18) | 0.004 | -0.41 (-0.47 to -0.34) | <0.001 |
| Memory IES | -3.63 (-4.88 to -2.38) | <0.001 |  | -0.55 (-1.51 to 0.42) | 0.267 | -0.46 (-0.53 to -0.39) | <0.001 |  | -0.38 (-1.32 to 0.56) | 0.431 | -0.44 (-0.51 to -0.38) | <0.001 |
| SDMT | 0.57 (0.49 to 0.65) | <0.001 |  | 0.24 (0.12 to 0.36) | <0.001 | -0.33 (-0.42 to -0.24) | <0.001 |  | 0.22 (0.10 to 0.33) | <0.001 | -0.33 (-0.42 to -0.24) | <0.001 |
| Stroop colour | 0.42 (0.35 to 0.50) | <0.001 |  | 0.10 (0.01 to 0.20) | 0.038 | -0.40 (-0.49 to -0.31) | <0.001 |  | 0.08 (-0.02 to 0.18) | 0.106 | -0.40 (-0.49 to -0.31) | <0.001 |
| Stroop words | 0.32 (0.26 to 0.38) | <0.001 |  | 0.09 (0.02 to 0.16) | 0.013 | -0.39 (-0.48 to -0.30) | <0.001 |  | 0.07 (0.00 to 0.14) | 0.049 | -0.39 (-0.48 to -0.30) | <0.001 |
| Stroop Interference | 0.60 (0.49 to 0.72) | <0.001 |  | 0.17 (0.05 to 0.30) | 0.008 | -0.4 (-0.48 to -0.32) | <0.001 |  | 0.13 (0.00 to 0.26) | 0.054 | -0.40 (-0.48 to -0.32) | <0.001 |
| Verbal fluency | 0.54 (0.42 to 0.65) | <0.001 |  | 0.18 (0.07 to 0.28) | 0.002 | -0.41 (-0.48 to -0.33) | <0.001 |  | 0.15 (0.04 to 0.26) | 0.006 | -0.39 (-0.47 to -0.32) | <0.001 |
| *Total Motor Score* | *-0.48 (0.10 to 0.18)* | *<0.001* |  |  |  |  |  |  |  |  |  |  |
| **Functional Checklist Score 0** | |  |  |  |  |  |  |  |  |  |  |  |
| Global IES | -1.47 (-1.8 to -1.15) | <0.001 |  | -0.6 (-0.94 to -0.26) | 0.001 | -0.11 (-0.13 to -0.08) | <0.001 |  | -0.60 (-0.94 to -0.26) | 0.001 | -0.11 (-0.13 to -0.08) | <0.001 |
| Executive IES | -2.17 (-2.73 to -1.6) | <0.001 |  | -0.78 (-1.31 to -0.24) | 0.005 | -0.12 (-0.14 to -0.09) | <0.001 |  | -0.83 (-1.37 to -0.29) | 0.003 | -0.11 (-0.14 to -0.09) | <0.001 |
| Visual IES | -1.33 (-1.79 to -0.86) | <0.001 |  | -0.44 (-0.83 to -0.06) | 0.025 | -0.12 (-0.15 to -0.1) | <0.001 |  | -0.45 (-0.84 to -0.07) | 0.021 | -0.12 (-0.14 to -0.10) | <0.001 |
| Language IES | -0.54 (-0.68 to -0.40) | <0.001 |  | -0.22 (-0.35 to -0.08) | 0.001 | -0.11 (-0.14 to -0.09) | <0.001 |  | -0.21 (-0.34 to -0.08) | 0.001 | -0.11 (-0.14 to -0.09) | <0.001 |
| Memory IES | -1.06 (-1.45 to -0.67) | <0.001 |  | -0.21 (-0.54 to 0.12) | 0.215 | -0.13 (-0.15 to -0.1) | <0.001 |  | -0.18 (-0.51 to 0.14) | 0.268 | -0.13 (-0.15 to -0.10) | <0.001 |
| SDMT | 0.16 (0.13 to 0.19) | <0.001 |  | 0.06 (0.02 to 0.11) | 0.002 | -0.09 (-0.13 to -0.06) | <0.001 |  | 0.06 (0.02 to 0.10) | 0.003 | -0.10 (-0.13 to -0.06) | <0.001 |
| Stroop colour | 0.12 (0.09 to 0.14) | <0.001 |  | 0.03 (0.00 to 0.06) | 0.079 | -0.11 (-0.14 to -0.08) | <0.001 |  | 0.03 (-0.01 to 0.06) | 0.112 | -0.11 (-0.14 to -0.08) | <0.001 |
| Stroop words | 0.09 (0.07 to 0.11) | <0.001 |  | 0.03 (0.01 to 0.06) | 0.01 | -0.1 (-0.13 to -0.07) | <0.001 |  | 0.03 (0.00 to 0.05) | 0.027 | -0.11 (-0.14 to -0.08) | <0.001 |
| Stroop Interference | 0.16 (0.12 to 0.20) | <0.001 |  | 0.03 (-0.01 to 0.08) | 0.167 | -0.12 (-0.15 to -0.09) | <0.001 |  | 0.02 (-0.02 to 0.07) | 0.321 | -0.12 (-0.15 to -0.09) | <0.001 |
| Verbal fluency | 0.14 (0.1 to 0.18) | <0.001 |  | 0.03 (-0.01 to 0.07) | 0.093 | -0.12 (-0.15 to -0.1) | <0.001 |  | 0.02 (-0.02 to 0.06) | 0.28 | -0.12 (-0.15 to -0.10) | <0.001 |
| *Total Motor Score* | *-0.13 (0.1 to 0.18)* | *<0.001* |  |  |  |  |  |  |  |  |  |  |

**Table S2. Association of raw scores of cognitive test and Total motor score with functional measures**

*Type 1 models: Results of linear univariate models. Type 2 models: Results of linear models adjusted for total motor score. Type 3 models: Results of linear models adjusted for total motor score, age at visit and years of education.*

|  | **Type 1 models** | |  | **Type 2 models** | | | |  | **Type 3 models** | | | |
| --- | --- | --- | --- | --- | --- | --- | --- | --- | --- | --- | --- | --- |
|  |  |  |  | **Cognitive Score** | | **Motor IES** | |  | **Cognitive Score** | | **Motor IES** | |
|  | **Coefficient (95%IC)** | **p-value** |  | **Coefficient (95%IC)** | **p-value** | **Coefficient (95%IC)** | **p-value** |  | **Coefficient (95%IC)** | **p-value** | **Coefficient (95%IC)** | **p-value** |
| **Total Functional Score** |  |  |  |  |  |  |  |  |  |  |  |  |
| Global IES | -2.16 (-2.7 to -1.61) | <0.001 |  | -1.52 (-2.14 to -0.9) | <0.001 | -5.19 (-7.88 to -2.5) | <0.001 |  | -1.43 (-2.05 to -0.82) | <0.001 | -4.59 (-7.33 to -1.85) | 0.001 |
| Executive IES | -3.44 (-4.35 to -2.53) | <0.001 |  | -2.41 (-3.39 to -1.42) | <0.001 | -5.65 (-8.25 to -3.05) | <0.001 |  | -2.31 (-3.3 to -1.31) | <0.001 | -5.14 (-7.78 to -2.5) | <0.001 |
| Visual IES | -1.94 (-2.69 to -1.19) | <0.001 |  | -1.07 (-1.83 to -0.3) | 0.006 | -7.19 (-9.81 to -4.56) | <0.001 |  | -0.97 (-1.73 to -0.22) | 0.012 | -6.33 (-9.04 to -3.62) | <0.001 |
| Language IES | -0.76 (-0.99 to -0.52) | <0.001 |  | -0.48 (-0.72 to -0.23) | <0.001 | -6.29 (-8.94 to -3.63) | <0.001 |  | -0.45 (-0.69 to -0.21) | <0.001 | -5.39 (-8.14 to -2.65) | <0.001 |
| Memory IES | -1.68 (-2.3 to -1.06) | <0.001 |  | -1.08 (-1.68 to -0.48) | 0.001 | -7.15 (-9.66 to -4.65) | <0.001 |  | -0.97 (-1.57 to -0.37) | 0.002 | -6.38 (-8.99 to -3.77) | <0.001 |
| Motor IES | -8.73 (-3.65 to -1.7) | <0.001 |  |  |  |  |  |  |  |  |  |  |
| SDMT | -1.56 (-1.82 to -1.31) | <0.001 |  | -1.42 (-1.75 to -1.09) | <0.001 | -1.77 (-4.34 to 0.8) | 0.176 |  | -1.38 (-1.7 to -1.05) | <0.001 | -1.49 (-4.09 to 1.1) | 0.257 |
| Stroop colour | -1.5 (-1.77 to -1.23) | <0.001 |  | -1.31 (-1.65 to -0.97) | <0.001 | -2.41 (-5.04 to 0.23) | 0.073 |  | -1.24 (-1.58 to -0.9) | <0.001 | -1.93 (-4.63 to 0.77) | 0.159 |
| Stroop words | -1.47 (-1.74 to -1.2) | <0.001 |  | -1.28 (-1.62 to -0.94) | <0.001 | -2.45 (-5.15 to 0.24) | 0.074 |  | -1.22 (-1.57 to -0.88) | <0.001 | -2.22 (-4.94 to 0.49) | 0.108 |
| Stroop Interference | -1.38 (-1.66 to -1.1) | <0.001 |  | -1.11 (-1.44 to -0.77) | <0.001 | -3.91 (-6.51 to -1.32) | 0.003 |  | -1.03 (-1.38 to -0.69) | <0.001 | -3.81 (-6.45 to -1.17) | 0.005 |
| Verbal fluency | -1.19 (-1.5 to -0.89) | <0.001 |  | -0.87 (-1.19 to -0.54) | <0.001 | -5.64 (-8.16 to -3.12) | <0.001 |  | -0.84 (-1.16 to -0.52) | <0.001 | -4.41 (-7.09 to -1.73) | 0.001 |
| Total motor score | -1.68 (-2.28 to -1.3) | <0.001 |  |  |  |  |  |  |  |  |  |  |
| **Independence scale** |  |  |  |  |  |  |  |  |  |  |  |  |
| Global IES | -12.46 (-15.13 to -9.79) | <0.001 |  | -9.04 (-12.04 to -6.04) | <0.001 | -27.85 (-40.93 to -14.77) | <0.001 |  | -8.55 (-11.52 to -5.58) | <0.001 | -24.62 (-37.86 to -11.37) | <0.001 |
| Executive IES | -18.15 (-22.81 to -13.5) | <0.001 |  | -12.01 (-16.95 to -7.07) | <0.001 | -33.54 (-46.6 to -20.48) | <0.001 |  | -11.34 (-16.31 to -6.38) | <0.001 | -30.44 (-43.63 to -17.24) | <0.001 |
| Visual IES | -11.35 (-15.14 to -7.56) | <0.001 |  | -6.55 (-10.32 to -2.77) | 0.001 | -39.42 (-52.43 to -26.41) | <0.001 |  | -6.04 (-9.76 to -2.33) | 0.002 | -34.72 (-48.05 to -21.39) | <0.001 |
| Language IES | -4.53 (-5.67 to -3.39) | <0.001 |  | -3.06 (-4.26 to -1.86) | <0.001 | -33.22 (-46.14 to -20.3) | <0.001 |  | -2.91 (-4.08 to -1.74) | <0.001 | -28.34 (-41.58 to -15.1) | <0.001 |
| Memory IES | -9.16 (-12.31 to -6.01) | <0.001 |  | -5.76 (-8.76 to -2.76) | <0.001 | -40.49 (-52.99 to -27.98) | <0.001 |  | -5.15 (-8.11 to -2.18) | 0.001 | -36.05 (-49 to -23.09) | <0.001 |
| Motor IES | -48.89 (-3.65 to -1.7) | <0.001 |  |  |  |  |  |  |  |  |  |  |
| SDMT | -8.54 (-9.79 to -7.28) | <0.001 |  | -7.61 (-9.2 to -6.02) | <0.001 | -11.67 (-24.14 to 0.81) | 0.067 |  | -7.34 (-8.92 to -5.76) | <0.001 | -9.98 (-22.47 to 2.52) | 0.117 |
| Stroop colour | -7.77 (-9.14 to -6.4) | <0.001 |  | -6.33 (-8.04 to -4.63) | <0.001 | -18.32 (-31.71 to -4.94) | 0.008 |  | -5.93 (-7.63 to -4.23) | <0.001 | -15.41 (-29.06 to -1.77) | 0.027 |
| Stroop words | -7.81 (-9.17 to -6.44) | <0.001 |  | -6.42 (-8.15 to -4.7) | <0.001 | -17.36 (-30.88 to -3.85) | 0.012 |  | -6.08 (-7.81 to -4.35) | <0.001 | -15.79 (-29.34 to -2.23) | 0.023 |
| Stroop Interference | -7.48 (-8.89 to -6.07) | <0.001 |  | -5.81 (-7.44 to -4.17) | <0.001 | -23.58 (-36.41 to -10.75) | <0.001 |  | -5.39 (-7.08 to -3.71) | <0.001 | -22.76 (-35.78 to -9.73) | 0.001 |
| Verbal fluency | -6.88 (-8.37 to -5.39) | <0.001 |  | -5.1 (-6.66 to -3.55) | <0.001 | -30.66 (-42.87 to -18.46) | <0.001 |  | -5.01 (-6.56 to -3.45) | <0.001 | -23.59 (-36.45 to -10.72) | <0.001 |
| Total motor score | -9.15 (-2.28 to -1.3) | <0.001 |  |  |  |  |  |  |  |  |  |  |
| **Functional Checklist Score** |  |  |  |  |  |  |  |  |  |  |  |  |
| Global IES | -3.72 (-4.55 to -2.89) | <0.001 |  | -2.6 (-3.52 to -1.67) | <0.001 | -9.13 (-13.16 to -5.1) | <0.001 |  | -2.57 (-3.5 to -1.65) | <0.001 | -9.05 (-13.17 to -4.93) | <0.001 |
| Executive IES | -5.47 (-6.9 to -4.03) | <0.001 |  | -3.51 (-5.02 to -1.99) | <0.001 | -10.69 (-14.69 to -6.69) | <0.001 |  | -3.6 (-5.14 to -2.07) | <0.001 | -10.61 (-14.68 to -6.54) | <0.001 |
| Visual IES | -3.35 (-4.52 to -2.18) | <0.001 |  | -1.82 (-2.97 to -0.66) | 0.002 | -12.55 (-16.53 to -8.56) | <0.001 |  | -1.8 (-2.95 to -0.65) | 0.002 | -12.12 (-16.26 to -7.98) | <0.001 |
| Language IES | -1.36 (-1.71 to -1.01) | <0.001 |  | -0.89 (-1.26 to -0.52) | <0.001 | -10.6 (-14.56 to -6.64) | <0.001 |  | -0.87 (-1.24 to -0.51) | <0.001 | -10.19 (-14.31 to -6.07) | <0.001 |
| Memory IES | -2.68 (-3.65 to -1.7) | <0.001 |  | -1.6 (-2.52 to -0.68) | 0.001 | -12.85 (-16.68 to -9.01) | <0.001 |  | -1.52 (-2.44 to -0.6) | 0.001 | -12.53 (-16.55 to -8.5) | <0.001 |
| Motor IES | -15.18 (-3.65 to -1.7) | <0.001 |  |  |  |  |  |  |  |  |  |  |
| SDMT | -2.38 (-2.8 to -1.96) | <0.001 |  | -1.92 (-2.44 to -1.39) | <0.001 | -5.79 (-9.92 to -1.66) | 0.006 |  | -1.91 (-2.44 to -1.38) | <0.001 | -5.96 (-10.13 to -1.79) | 0.005 |
| Stroop colour | -2.25 (-2.69 to -1.81) | <0.001 |  | -1.7 (-2.24 to -1.16) | <0.001 | -6.96 (-11.19 to -2.74) | 0.001 |  | -1.62 (-2.17 to -1.08) | <0.001 | -7.01 (-11.38 to -2.64) | 0.002 |
| Stroop words | -2.2 (-2.64 to -1.75) | <0.001 |  | -1.62 (-2.17 to -1.07) | <0.001 | -7.23 (-11.56 to -2.9) | 0.001 |  | -1.6 (-2.16 to -1.04) | <0.001 | -7.41 (-11.78 to -3.03) | 0.001 |
| Stroop Interference | -1.99 (-2.45 to -1.52) | <0.001 |  | -1.32 (-1.85 to -0.79) | <0.001 | -9.43 (-13.6 to -5.26) | <0.001 |  | -1.28 (-1.83 to -0.72) | <0.001 | -9.75 (-14 to -5.5) | <0.001 |
| Verbal fluency | -1.79 (-2.28 to -1.3) | <0.001 |  | -1.14 (-1.65 to -0.64) | <0.001 | -11.09 (-15.02 to -7.15) | <0.001 |  | -1.07 (-1.58 to -0.56) | <0.001 | -10.37 (-14.62 to -6.12) | <0.001 |
| Total motor score | -2.56 (-2.28 to -1.3) | <0.001 |  |  |  |  |  |  |  |  |  |  |

**Table S3. Association of normalized scores cognitive test and motor IES with and functional measures**

*Type 1 models: Results of linear univariate models. Type 2 models: Results of linear models adjusted for Motor IES. Type 3 models: Results of linear models adjusted for Motor IES, age at visit and years of education. IES was normalized using the overall mean and standard deviation of all scores together by the measure of functional limitation and the other test scores were normalized using the mean and standard deviation of each score by the measure of functional limitation, then, normalized scores were inverted (multiplied by -1) for comparison with the IES scores.*

|  | **Cognitive variable** | | | **Motor IES** | | | **Apathy Score** | | |
| --- | --- | --- | --- | --- | --- | --- | --- | --- | --- |
|  | **Coefficient (95%IC)** | **p-value** | **Tolerance** | **Coefficient (95%IC)** | **p-value** | **Tolerance** | **Coefficient (95%IC)** | **p-value** | **Tolerance** |
| **Total Functional Score** |  |  |  |  |  |  |  |  |  |
| Global IES | -1.16 (-1.77 to -0.56) | <0.001 | 0.67 | -4.73 (-7.35 to -2.11) | <0.001 | 0.66 | -0.2 (-0.28 to -0.11) | <0.001 | 0.94 |
| Executive IES | -1.9 (-2.89 to -0.92) | <0.001 | 0.67 | -5.14 (-7.67 to -2.61) | <0.001 | 0.71 | -0.19 (-0.28 to -0.11) | <0.001 | 0.92 |
| Visual IES | -0.97 (-1.68 to -0.27) | 0.007 | 0.81 | -5.85 (-8.39 to -3.32) | <0.001 | 0.74 | -0.23 (-0.32 to -0.14) | <0.001 | 0.97 |
| Language IES | -0.34 (-0.58 to -0.11) | 0.005 | 0.74 | -5.48 (-8.1 to -2.87) | <0.001 | 0.7 | -0.21 (-0.3 to -0.12) | <0.001 | 0.94 |
| Memory IES | -0.76 (-1.34 to -0.18) | 0.01 | 0.83 | -6.2 (-8.67 to -3.72) | <0.001 | 0.78 | -0.21 (-0.3 to -0.12) | <0.001 | 0.95 |
| SDMT | -1.19 (-1.54 to -0.85) | <0.001 | 0.53 | -1.93 (-4.51 to 0.66) | 0.143 | 0.57 | -0.15 (-0.23 to -0.06) | 0.001 | 0.89 |
| Stroop colour | -1.1 (-1.44 to -0.76) | <0.001 | 0.56 | -2.23 (-4.86 to 0.39) | 0.095 | 0.57 | -0.19 (-0.27 to -0.11) | <0.001 | 0.95 |
| Stroop words | -1.1 (-1.43 to -0.76) | <0.001 | 0.57 | -2.06 (-4.7 to 0.58) | 0.125 | 0.56 | -0.18 (-0.26 to -0.1) | <0.001 | 0.94 |
| Stroop Interference | -0.86 (-1.2 to -0.52) | <0.001 | 0.61 | -4 (-6.56 to -1.44) | 0.002 | 0.65 | -0.19 (-0.27 to -0.1) | <0.001 | 0.93 |
| Verbal fluency | -0.63 (-0.97 to -0.29) | <0.001 | 0.65 | -4.75 (-7.4 to -2.11) | 0.001 | 0.66 | -0.18 (-0.27 to -0.09) | <0.001 | 0.87 |
| **Independence scale** |  |  |  |  |  |  |  |  |  |
| Global IES | -7.22 (-10.1 to -4.34) | <0.001 | 0.67 | -25.39 (-37.92 to -12.85) | <0.001 | 0.66 | -1.01 (-1.43 to -0.6) | <0.001 | 0.94 |
| Executive IES | -9.07 (-13.93 to -4.21) | <0.001 | 0.67 | -30.71 (-43.21 to -18.21) | <0.001 | 0.71 | -1.03 (-1.47 to -0.6) | <0.001 | 0.92 |
| Visual IES | -6.04 (-9.46 to -2.61) | 0.001 | 0.81 | -32.38 (-44.68 to -20.08) | <0.001 | 0.74 | -1.22 (-1.64 to -0.79) | <0.001 | 0.97 |
| Language IES | -2.39 (-3.52 to -1.26) | <0.001 | 0.74 | -28.86 (-41.35 to -16.36) | <0.001 | 0.7 | -1.05 (-1.47 to -0.63) | <0.001 | 0.94 |
| Memory IES | -4.08 (-6.91 to -1.26) | 0.005 | 0.83 | -35.23 (-47.36 to -23.11) | <0.001 | 0.78 | -1.12 (-1.55 to -0.68) | <0.001 | 0.95 |
| SDMT | -6.42 (-8.06 to -4.79) | <0.001 | 0.53 | -12.25 (-24.57 to 0.07) | 0.051 | 0.57 | -0.76 (-1.15 to -0.37) | <0.001 | 0.89 |
| Stroop colour | -5.43 (-7.11 to -3.75) | <0.001 | 0.56 | -16.08 (-29 to -3.15) | 0.015 | 0.57 | -1.02 (-1.41 to -0.62) | <0.001 | 0.95 |
| Stroop words | -5.16 (-6.83 to -3.49) | <0.001 | 0.57 | -16.37 (-29.52 to -3.22) | 0.015 | 0.56 | -0.98 (-1.38 to -0.59) | <0.001 | 0.94 |
| Stroop Interference | -4.49 (-6.15 to -2.84) | <0.001 | 0.61 | -23.83 (-36.29 to -11.36) | <0.001 | 0.65 | -0.99 (-1.4 to -0.58) | <0.001 | 0.93 |
| Verbal fluency | -3.93 (-5.55 to -2.3) | <0.001 | 0.65 | -25.53 (-38.2 to -12.87) | <0.001 | 0.66 | -0.88 (-1.31 to -0.45) | <0.001 | 0.87 |
| **Functional Checklist Score** |  |  |  |  |  |  |  |  |  |
| Global IES | -2.22 (-3.13 to -1.3) | <0.001 | 0.67 | -9.17 (-13.15 to -5.2) | <0.001 | 0.66 | -0.28 (-0.41 to -0.15) | <0.001 | 0.94 |
| Executive IES | -3.01 (-4.53 to -1.48) | <0.001 | 0.67 | -10.59 (-14.51 to -6.66) | <0.001 | 0.71 | -0.29 (-0.42 to -0.15) | <0.001 | 0.92 |
| Visual IES | -1.82 (-2.91 to -0.74) | 0.001 | 0.81 | -11.36 (-15.26 to -7.47) | <0.001 | 0.74 | -0.35 (-0.48 to -0.21) | <0.001 | 0.97 |
| Language IES | -0.73 (-1.09 to -0.38) | <0.001 | 0.74 | -10.24 (-14.2 to -6.28) | <0.001 | 0.7 | -0.29 (-0.43 to -0.16) | <0.001 | 0.94 |
| Memory IES | -1.21 (-2.1 to -0.31) | 0.009 | 0.83 | -12.25 (-16.09 to -8.41) | <0.001 | 0.78 | -0.32 (-0.45 to -0.18) | <0.001 | 0.95 |
| SDMT | -1.61 (-2.17 to -1.06) | <0.001 | 0.53 | -6.69 (-10.86 to -2.52) | 0.002 | 0.57 | -0.23 (-0.36 to -0.1) | 0.001 | 0.89 |
| Stroop colour | -1.4 (-1.95 to -0.84) | <0.001 | 0.56 | -7.51 (-11.77 to -3.24) | 0.001 | 0.57 | -0.29 (-0.42 to -0.16) | <0.001 | 0.95 |
| Stroop words | -1.38 (-1.93 to -0.84) | <0.001 | 0.57 | -7.32 (-11.62 to -3.03) | 0.001 | 0.56 | -0.28 (-0.41 to -0.15) | <0.001 | 0.94 |
| Stroop Interference | -0.99 (-1.53 to -0.44) | <0.001 | 0.61 | -10.1 (-14.23 to -5.97) | <0.001 | 0.65 | -0.29 (-0.43 to -0.16) | <0.001 | 0.93 |
| Verbal fluency | -0.75 (-1.28 to -0.21) | 0.007 | 0.65 | -10.91 (-15.1 to -6.72) | <0.001 | 0.66 | -0.28 (-0.42 to -0.14) | <0.001 | 0.87 |

**Table S4. Association of normalized scores cognitive test and motor IES with and functional measures adjusted to Apathy score of Problem Behaviors Assessment**

*Results of linear models adjusted for cognitive test, Motor IES, apathy score of PBA, age at visit, years of education. IES was normalized using the overall mean and standard deviation of all scores together by the measure of functional limitation and the other test scores were normalized using the mean and standard deviation of each score by the measure of functional limitation, then, normalized scores were inverted (multiplied by -1) for comparison with the IES scores.*

**Table S5. Association of normalized scores cognitive test and motor IES with and functional measures adjusted to Depression score of Problem Behaviors Assessment**

|  | **Cognitive variable** | | | **Motor IES** | | | **Depression Score** | | |
| --- | --- | --- | --- | --- | --- | --- | --- | --- | --- |
|  | **Coefficient (95%IC)** | **p-value** | **Tolerance** | **Coefficient (95%IC)** | **p-value** | **Tolerance** | **Coefficient (95%IC)** | **p-value** | **Tolerance** |
| **Total Functional Score** |  |  |  |  |  |  |  |  |  |
| Global IES | -1.42 (-2.06 to -0.79) | <0.001 | 0.69 | -4.64 (-7.44 to -1.84) | 0.001 | 0.66 | 0 (-0.06 to 0.06) | 0.978 | 0.96 |
| Executive IES | -2.39 (-3.42 to -1.37) | <0.001 | 0.7 | -5.06 (-7.75 to -2.37) | <0.001 | 0.71 | 0.01 (-0.05 to 0.07) | 0.814 | 0.95 |
| Visual IES | -0.98 (-1.75 to -0.21) | 0.013 | 0.8 | -6.38 (-9.13 to -3.63) | <0.001 | 0.75 | -0.02 (-0.08 to 0.05) | 0.636 | 0.95 |
| Language IES | -0.44 (-0.69 to -0.19) | 0.001 | 0.77 | -5.48 (-8.28 to -2.68) | <0.001 | 0.69 | 0 (-0.06 to 0.06) | 0.955 | 0.96 |
| Memory IES | -0.97 (-1.58 to -0.36) | 0.002 | 0.85 | -6.43 (-9.09 to -3.78) | <0.001 | 0.78 | 0 (-0.06 to 0.06) | 0.999 | 0.96 |
| SDMT | -1.38 (-1.72 to -1.04) | <0.001 | 0.58 | -1.43 (-4.11 to 1.26) | 0.295 | 0.57 | 0.01 (-0.04 to 0.06) | 0.719 | 0.96 |
| Stroop colour | -1.22 (-1.59 to -0.86) | <0.001 | 0.57 | -2.12 (-4.94 to 0.69) | 0.139 | 0.57 | 0 (-0.06 to 0.06) | 0.951 | 0.96 |
| Stroop words | -1.24 (-1.59 to -0.89) | <0.001 | 0.59 | -1.83 (-4.64 to 0.98) | 0.2 | 0.56 | 0 (-0.06 to 0.05) | 0.918 | 0.96 |
| Stroop Interference | -1.01 (-1.36 to -0.66) | <0.001 | 0.63 | -3.86 (-6.59 to -1.14) | 0.006 | 0.65 | 0 (-0.05 to 0.06) | 0.879 | 0.96 |
| Verbal fluency | -0.84 (-1.18 to -0.5) | <0.001 | 0.72 | -4.35 (-7.12 to -1.58) | 0.002 | 0.66 | 0 (-0.06 to 0.06) | 0.997 | 0.96 |
| **Independence scale** |  |  |  |  |  |  |  |  |  |
| Global IES | -8.48 (-11.52 to -5.45) | <0.001 | 0.69 | -25.11 (-38.56 to -11.66) | <0.001 | 0.66 | -0.14 (-0.43 to 0.15) | 0.342 | 0.96 |
| Executive IES | -11.43 (-16.54 to -6.32) | <0.001 | 0.7 | -30.6 (-44.01 to -17.19) | <0.001 | 0.71 | -0.11 (-0.41 to 0.19) | 0.474 | 0.95 |
| Visual IES | -6.24 (-10.01 to -2.47) | 0.001 | 0.8 | -34.97 (-48.42 to -21.52) | <0.001 | 0.75 | -0.23 (-0.54 to 0.08) | 0.146 | 0.95 |
| Language IES | -2.87 (-4.06 to -1.67) | <0.001 | 0.77 | -28.99 (-42.44 to -15.55) | <0.001 | 0.69 | -0.14 (-0.44 to 0.16) | 0.344 | 0.96 |
| Memory IES | -5.08 (-8.11 to -2.05) | 0.001 | 0.85 | -36.62 (-49.73 to -23.52) | <0.001 | 0.78 | -0.14 (-0.45 to 0.17) | 0.369 | 0.96 |
| SDMT | -7.33 (-8.97 to -5.69) | <0.001 | 0.58 | -9.97 (-22.84 to 2.89) | 0.128 | 0.57 | -0.09 (-0.35 to 0.17) | 0.503 | 0.96 |
| Stroop colour | -6.06 (-7.85 to -4.26) | <0.001 | 0.57 | -15.64 (-29.61 to -1.66) | 0.029 | 0.57 | -0.15 (-0.43 to 0.13) | 0.287 | 0.96 |
| Stroop words | -5.89 (-7.65 to -4.13) | <0.001 | 0.59 | -15.27 (-29.37 to -1.16) | 0.034 | 0.56 | -0.16 (-0.44 to 0.12) | 0.267 | 0.96 |
| Stroop Interference | -5.24 (-6.98 to -3.5) | <0.001 | 0.63 | -23.37 (-36.73 to -10) | 0.001 | 0.65 | -0.12 (-0.41 to 0.17) | 0.42 | 0.96 |
| Verbal fluency | -4.94 (-6.56 to -3.31) | <0.001 | 0.72 | -23.67 (-36.94 to -10.4) | 0.001 | 0.66 | -0.14 (-0.42 to 0.15) | 0.351 | 0.96 |
| **Functional Checklist Score** |  |  |  |  |  |  |  |  |  |
| Global IES | -2.6 (-3.55 to -1.64) | <0.001 | 0.69 | -9.04 (-13.25 to -4.83) | <0.001 | 0.66 | 0.01 (-0.08 to 0.1) | 0.849 | 0.96 |
| Executive IES | -3.75 (-5.33 to -2.16) | <0.001 | 0.7 | -10.45 (-14.61 to -6.3) | <0.001 | 0.71 | 0.02 (-0.07 to 0.11) | 0.677 | 0.95 |
| Visual IES | -1.82 (-3 to -0.64) | 0.003 | 0.8 | -12.16 (-16.37 to -7.95) | <0.001 | 0.75 | -0.02 (-0.12 to 0.08) | 0.719 | 0.95 |
| Language IES | -0.88 (-1.25 to -0.5) | <0.001 | 0.77 | -10.22 (-14.43 to -6.02) | <0.001 | 0.69 | 0.01 (-0.09 to 0.1) | 0.865 | 0.96 |
| Memory IES | -1.52 (-2.47 to -0.58) | 0.002 | 0.85 | -12.6 (-16.69 to -8.5) | <0.001 | 0.78 | 0.01 (-0.09 to 0.11) | 0.863 | 0.96 |
| SDMT | -1.91 (-2.46 to -1.36) | <0.001 | 0.58 | -5.89 (-10.21 to -1.57) | 0.008 | 0.57 | 0.02 (-0.07 to 0.11) | 0.639 | 0.96 |
| Stroop colour | -1.59 (-2.17 to -1) | <0.001 | 0.57 | -7.33 (-11.88 to -2.78) | 0.002 | 0.57 | 0 (-0.09 to 0.1) | 0.928 | 0.96 |
| Stroop words | -1.6 (-2.17 to -1.03) | <0.001 | 0.59 | -6.96 (-11.51 to -2.41) | 0.003 | 0.56 | 0 (-0.09 to 0.09) | 0.954 | 0.96 |
| Stroop Interference | -1.23 (-1.8 to -0.66) | <0.001 | 0.63 | -9.88 (-14.26 to -5.5) | <0.001 | 0.65 | 0.01 (-0.08 to 0.11) | 0.812 | 0.96 |
| Verbal fluency | -1.08 (-1.62 to -0.55) | <0.001 | 0.72 | -10.25 (-14.64 to -5.86) | <0.001 | 0.66 | 0.01 (-0.09 to 0.1) | 0.892 | 0.96 |

*Results of linear models adjusted for cognitive test, Motor IES, depression score of PBA, age at visit, years of education. IES was normalized using the overall mean and standard deviation of all scores together by the measure of functional limitation and the other test scores were normalized using the mean and standard deviation of each score by the measure of functional limitation, then, normalized scores were inverted (multiplied by -1) for comparison with the IES scores.*

|  | **Cognitive variable** | | | **Motor variable** | | | **Irritability Score** | | |
| --- | --- | --- | --- | --- | --- | --- | --- | --- | --- |
|  | **Coefficient (95%IC)** | **p-value** | **Tolerance** | **Coefficient (95%IC)** | **p-value** | **Tolerance** | **Coefficient (95%IC)** | **p-value** | **Tolerance** |
| **Total Functional Score** |  |  |  |  |  |  |  |  |  |
| Global IES | -1.51 (-2.1 to -0.91) | <0.001 | 0.69 | -4.53 (-7.16 to -1.91) | 0.001 | 0.66 | -0.19 (-0.27 to -0.11) | <0.001 | 0.98 |
| Executive IES | -2.31 (-3.28 to -1.34) | <0.001 | 0.7 | -5.2 (-7.76 to -2.65) | <0.001 | 0.71 | -0.17 (-0.25 to -0.09) | <0.001 | 0.99 |
| Visual IES | -1.07 (-1.8 to -0.34) | 0.004 | 0.8 | -6.33 (-8.93 to -3.72) | <0.001 | 0.75 | -0.18 (-0.27 to -0.1) | <0.001 | 0.98 |
| Language IES | -0.49 (-0.73 to -0.26) | <0.001 | 0.76 | -5.29 (-7.92 to -2.67) | <0.001 | 0.69 | -0.19 (-0.28 to -0.11) | <0.001 | 0.98 |
| Memory IES | -0.92 (-1.5 to -0.33) | 0.002 | 0.85 | -6.55 (-9.08 to -4.02) | <0.001 | 0.78 | -0.17 (-0.26 to -0.08) | <0.001 | 0.99 |
| SDMT | -1.28 (-1.62 to -0.95) | <0.001 | 0.56 | -1.91 (-4.51 to 0.7) | 0.15 | 0.57 | -0.13 (-0.2 to -0.05) | 0.002 | 0.96 |
| Stroop colour | -1.14 (-1.49 to -0.79) | <0.001 | 0.56 | -2.55 (-5.27 to 0.17) | 0.066 | 0.56 | -0.14 (-0.22 to -0.06) | 0.001 | 0.97 |
| Stroop words | -1.13 (-1.48 to -0.79) | <0.001 | 0.57 | -2.35 (-5.1 to 0.39) | 0.092 | 0.55 | -0.13 (-0.21 to -0.04) | 0.003 | 0.95 |
| Stroop Interference | -0.92 (-1.26 to -0.57) | <0.001 | 0.62 | -4.26 (-6.89 to -1.62) | 0.002 | 0.65 | -0.14 (-0.22 to -0.06) | 0.001 | 0.96 |
| Verbal fluency | -0.77 (-1.1 to -0.44) | <0.001 | 0.71 | -4.68 (-7.34 to -2.01) | 0.001 | 0.66 | -0.15 (-0.24 to -0.07) | 0.001 | 0.97 |
| **Independence scale** |  |  |  |  |  |  |  |  |  |
| Global IES | -8.82 (-11.78 to -5.85) | <0.001 | 0.69 | -24.59 (-37.7 to -11.48) | <0.001 | 0.66 | -0.62 (-1.03 to -0.2) | 0.004 | 0.98 |
| Executive IES | -11.41 (-16.42 to -6.41) | <0.001 | 0.7 | -30.76 (-43.94 to -17.58) | <0.001 | 0.71 | -0.51 (-0.94 to -0.08) | 0.021 | 0.99 |
| Visual IES | -6.31 (-10.01 to -2.61) | 0.001 | 0.8 | -35.02 (-48.25 to -21.79) | <0.001 | 0.75 | -0.6 (-1.04 to -0.15) | 0.009 | 0.98 |
| Language IES | -3.07 (-4.24 to -1.9) | <0.001 | 0.76 | -28.21 (-41.3 to -15.13) | <0.001 | 0.69 | -0.65 (-1.07 to -0.23) | 0.003 | 0.98 |
| Memory IES | -5.03 (-8.01 to -2.04) | 0.001 | 0.85 | -36.83 (-49.74 to -23.91) | <0.001 | 0.78 | -0.51 (-0.95 to -0.07) | 0.025 | 0.99 |
| SDMT | -7.18 (-8.83 to -5.52) | <0.001 | 0.56 | -10.71 (-23.57 to 2.15) | 0.102 | 0.57 | -0.26 (-0.64 to 0.12) | 0.175 | 0.96 |
| Stroop colour | -5.87 (-7.67 to -4.07) | <0.001 | 0.56 | -16.57 (-30.51 to -2.63) | 0.02 | 0.56 | -0.36 (-0.77 to 0.05) | 0.086 | 0.97 |
| Stroop words | -5.67 (-7.46 to -3.88) | <0.001 | 0.57 | -16.35 (-30.52 to -2.17) | 0.024 | 0.55 | -0.29 (-0.7 to 0.13) | 0.171 | 0.95 |
| Stroop Interference | -5.06 (-6.8 to -3.31) | <0.001 | 0.62 | -24.12 (-37.43 to -10.8) | <0.001 | 0.65 | -0.35 (-0.77 to 0.07) | 0.101 | 0.96 |
| Verbal fluency | -4.79 (-6.41 to -3.17) | <0.001 | 0.71 | -24.35 (-37.54 to -11.16) | <0.001 | 0.66 | -0.39 (-0.8 to 0.03) | 0.068 | 0.97 |
| **Functional Checklist Score** | |  |  |  |  |  |  |  |  |
| Global IES | -2.65 (-3.59 to -1.71) | <0.001 | 0.69 | -8.97 (-13.13 to -4.82) | <0.001 | 0.66 | -0.13 (-0.26 to 0) | 0.048 | 0.98 |
| Executive IES | -3.67 (-5.23 to -2.1) | <0.001 | 0.7 | -10.57 (-14.69 to -6.45) | <0.001 | 0.71 | -0.1 (-0.23 to 0.04) | 0.148 | 0.99 |
| Visual IES | -1.88 (-3.04 to -0.71) | 0.002 | 0.8 | -12.13 (-16.3 to -7.96) | <0.001 | 0.75 | -0.13 (-0.27 to 0.01) | 0.076 | 0.98 |
| Language IES | -0.91 (-1.28 to -0.54) | <0.001 | 0.76 | -10.1 (-14.24 to -5.95) | <0.001 | 0.69 | -0.14 (-0.28 to -0.01) | 0.038 | 0.98 |
| Memory IES | -1.49 (-2.43 to -0.55) | 0.002 | 0.85 | -12.67 (-16.75 to -8.6) | <0.001 | 0.78 | -0.1 (-0.24 to 0.04) | 0.158 | 0.99 |
| SDMT | -1.87 (-2.43 to -1.32) | <0.001 | 0.56 | -6.07 (-10.41 to -1.74) | 0.006 | 0.57 | -0.04 (-0.17 to 0.09) | 0.567 | 0.96 |
| Stroop colour | -1.55 (-2.14 to -0.96) | <0.001 | 0.56 | -7.52 (-12.08 to -2.97) | 0.001 | 0.56 | -0.06 (-0.2 to 0.07) | 0.365 | 0.97 |
| Stroop words | -1.57 (-2.15 to -0.99) | <0.001 | 0.57 | -7.13 (-11.72 to -2.55) | 0.002 | 0.55 | -0.04 (-0.17 to 0.09) | 0.552 | 0.95 |
| Stroop Interference | -1.18 (-1.76 to -0.61) | <0.001 | 0.62 | -10.08 (-14.46 to -5.7) | <0.001 | 0.65 | -0.07 (-0.2 to 0.07) | 0.35 | 0.96 |
| Verbal fluency | -1.04 (-1.58 to -0.51) | <0.001 | 0.71 | -10.43 (-14.8 to -6.05) | <0.001 | 0.66 | -0.08 (-0.21 to 0.06) | 0.276 | 0.97 |

**Table S6. Association of normalized scores cognitive test and motor IES with and functional measures adjusted to Irritability score of Problem Behaviors Assessment**

*Results of linear models adjusted for cognitive test, Motor IES, irritability score of PBA, age at visit, years of education. IES was normalized using the overall mean and standard deviation of all scores together by the measure of functional limitation and the other test scores were normalized using the mean and standard deviation of each score by the measure of functional limitation, then, normalized scores were inverted (multiplied by -1) for comparison with the IES scores.*

**Table S7. Association of normalized scores cognitive test and motor IES with and functional measures adjusted to Executive functions/ Obsessive-compulsive score of Problem Behaviors Assessment**

|  | **Cognitive variable** | | | **Motor variable** | | | **Executive function Score** | | |
| --- | --- | --- | --- | --- | --- | --- | --- | --- | --- |
|  | **Coefficient (95%IC)** | **p-value** | **Tolerance** | **Coefficient (95%IC)** | **p-value** | **Tolerance** | **Coefficient (95%IC)** | **p-value** | **Tolerance** |
| **Total Functional Score** |  |  |  |  |  |  |  |  |  |
| Global IES | -1.29 (-1.88 to -0.7) | <0.001 | 0.69 | -4.2 (-6.81 to -1.6) | 0.002 | 0.66 | -0.2 (-0.28 to -0.12) | <0.001 | 0.97 |
| Executive IES | -2.01 (-2.98 to -1.04) | <0.001 | 0.68 | -4.79 (-7.31 to -2.26) | <0.001 | 0.71 | -0.19 (-0.27 to -0.11) | <0.001 | 0.95 |
| Visual IES | -0.86 (-1.57 to -0.14) | 0.019 | 0.8 | -5.77 (-8.33 to -3.21) | <0.001 | 0.74 | -0.21 (-0.3 to -0.13) | <0.001 | 0.98 |
| Language IES | -0.4 (-0.63 to -0.17) | 0.001 | 0.76 | -4.94 (-7.54 to -2.34) | <0.001 | 0.69 | -0.21 (-0.29 to -0.12) | <0.001 | 0.98 |
| Memory IES | -0.93 (-1.49 to -0.36) | 0.001 | 0.85 | -5.72 (-8.18 to -3.25) | <0.001 | 0.77 | -0.21 (-0.3 to -0.13) | <0.001 | 0.98 |
| SDMT | -1.2 (-1.54 to -0.86) | <0.001 | 0.53 | -1.75 (-4.33 to 0.84) | 0.183 | 0.57 | -0.14 (-0.22 to -0.06) | 0.001 | 0.9 |
| Stroop colour | -1.06 (-1.41 to -0.71) | <0.001 | 0.54 | -2.27 (-4.94 to 0.41) | 0.096 | 0.57 | -0.16 (-0.24 to -0.08) | <0.001 | 0.93 |
| Stroop words | -1.09 (-1.43 to -0.74) | <0.001 | 0.56 | -1.95 (-4.61 to 0.71) | 0.15 | 0.56 | -0.16 (-0.24 to -0.09) | <0.001 | 0.94 |
| Stroop Interference | -0.84 (-1.18 to -0.49) | <0.001 | 0.6 | -3.89 (-6.47 to -1.3) | 0.003 | 0.65 | -0.17 (-0.25 to -0.09) | <0.001 | 0.93 |
| Verbal fluency | -0.71 (-1.03 to -0.38) | <0.001 | 0.7 | -4.21 (-6.81 to -1.6) | 0.002 | 0.66 | -0.18 (-0.27 to -0.1) | <0.001 | 0.95 |
| **Independence scale** |  |  |  |  |  |  |  |  |  |
| Global IES | -7.79 (-10.55 to -5.03) | <0.001 | 0.69 | -22.49 (-34.69 to -10.29) | <0.001 | 0.66 | -1.13 (-1.51 to -0.74) | <0.001 | 0.97 |
| Executive IES | -9.49 (-14.22 to -4.76) | <0.001 | 0.68 | -28.72 (-41.03 to -16.42) | <0.001 | 0.71 | -1.09 (-1.5 to -0.69) | <0.001 | 0.95 |
| Visual IES | -5.38 (-8.8 to -1.96) | 0.002 | 0.8 | -31.73 (-44.02 to -19.44) | <0.001 | 0.74 | -1.19 (-1.6 to -0.78) | <0.001 | 0.98 |
| Language IES | -2.66 (-3.74 to -1.57) | <0.001 | 0.76 | -25.83 (-38.02 to -13.63) | <0.001 | 0.69 | -1.16 (-1.55 to -0.76) | <0.001 | 0.98 |
| Memory IES | -4.95 (-7.67 to -2.22) | <0.001 | 0.85 | -32.43 (-44.32 to -20.54) | <0.001 | 0.77 | -1.21 (-1.61 to -0.8) | <0.001 | 0.98 |
| SDMT | -6.34 (-7.96 to -4.72) | <0.001 | 0.53 | -11.5 (-23.68 to 0.67) | 0.064 | 0.57 | -0.8 (-1.17 to -0.43) | <0.001 | 0.9 |
| Stroop colour | -5.12 (-6.84 to -3.41) | <0.001 | 0.54 | -16.32 (-29.32 to -3.32) | 0.014 | 0.57 | -0.97 (-1.36 to -0.58) | <0.001 | 0.93 |
| Stroop words | -5.01 (-6.68 to -3.33) | <0.001 | 0.56 | -15.84 (-28.93 to -2.74) | 0.018 | 0.56 | -0.98 (-1.37 to -0.59) | <0.001 | 0.94 |
| Stroop Interference | -4.31 (-5.98 to -2.65) | <0.001 | 0.6 | -23.24 (-35.67 to -10.82) | <0.001 | 0.65 | -0.98 (-1.38 to -0.58) | <0.001 | 0.93 |
| Verbal fluency | -4.23 (-5.75 to -2.7) | <0.001 | 0.7 | -22.71 (-34.95 to -10.48) | <0.001 | 0.66 | -1.03 (-1.42 to -0.63) | <0.001 | 0.95 |
| **Funcitonal Checklist Score** |  |  |  |  |  |  |  |  |  |
| Global IES | -2.43 (-3.35 to -1.52) | <0.001 | 0.69 | -8.54 (-12.58 to -4.5) | <0.001 | 0.66 | -0.23 (-0.36 to -0.11) | <0.001 | 0.97 |
| Executive IES | -3.28 (-4.83 to -1.74) | <0.001 | 0.68 | -10.16 (-14.18 to -6.15) | <0.001 | 0.71 | -0.22 (-0.35 to -0.09) | 0.001 | 0.95 |
| Visual IES | -1.68 (-2.8 to -0.55) | 0.004 | 0.8 | -11.43 (-15.47 to -7.38) | <0.001 | 0.74 | -0.25 (-0.39 to -0.12) | <0.001 | 0.98 |
| Language IES | -0.83 (-1.18 to -0.47) | <0.001 | 0.76 | -9.6 (-13.64 to -5.57) | <0.001 | 0.69 | -0.24 (-0.37 to -0.11) | <0.001 | 0.98 |
| Memory IES | -1.47 (-2.37 to -0.57) | 0.002 | 0.85 | -11.74 (-15.67 to -7.8) | <0.001 | 0.77 | -0.26 (-0.39 to -0.13) | <0.001 | 0.98 |
| SDMT | -1.71 (-2.27 to -1.14) | <0.001 | 0.53 | -6.27 (-10.52 to -2.01) | 0.004 | 0.57 | -0.15 (-0.28 to -0.02) | 0.024 | 0.9 |
| Stroop colour | -1.39 (-1.98 to -0.81) | <0.001 | 0.54 | -7.5 (-11.93 to -3.08) | 0.001 | 0.57 | -0.19 (-0.33 to -0.06) | 0.004 | 0.93 |
| Stroop words | -1.42 (-1.99 to -0.85) | <0.001 | 0.56 | -7.11 (-11.53 to -2.68) | 0.002 | 0.56 | -0.2 (-0.33 to -0.06) | 0.004 | 0.94 |
| Stroop Interference | -1.02 (-1.59 to -0.45) | 0.001 | 0.6 | -9.92 (-14.17 to -5.67) | <0.001 | 0.65 | -0.21 (-0.34 to -0.07) | 0.003 | 0.93 |
| Verbal fluency | -0.92 (-1.45 to -0.39) | 0.001 | 0.7 | -10.09 (-14.33 to -5.85) | <0.001 | 0.66 | -0.22 (-0.36 to -0.09) | 0.002 | 0.95 |

*Results of linear models adjusted for cognitive test, Motor IES, executive function score of PBA-s, age at visit, years of education. IES was normalized using the overall mean and standard deviation of all scores together by the measure of functional limitation and the other test scores were normalized using the mean and standard deviation of each score by the measure of functional limitation, then, normalized scores were inverted (multiplied by -1) for comparison with the IES scores.*

**Table S8. Association of normalized scores cognitive test and motor IES with and functional measures adjusted to for antidepressant**

|  | **Cognitive variable** | | | **Motor IES** | | | **Antidepressant exposure** | | |
| --- | --- | --- | --- | --- | --- | --- | --- | --- | --- |
| **Total Functional Score** | **Coefficient (95%IC)** | **p-value** | **Tolerance** | **Coefficient (95%IC)** | **p-value** | **Tolerance** | **Coefficient (95%IC)** | **p-value** | **Tolerance** |
| Global IES | -1.56 (-2.13 to -0.99) | <0.001 | 0.69 | -3.51 (-6.03 to -0.99) | 0.007 | 0.65 | -0.74 (-1.39 to -0.09) | 0.026 | 0.86 |
| Executive IES | -1.62 (-2.53 to -0.71) | 0.001 | 0.79 | -5.01 (-7.58 to -2.43) | <0.001 | 0.71 | -1 (-1.68 to -0.32) | 0.004 | 0.89 |
| Visual IES | -0.54 (-0.77 to -0.31) | <0.001 | 0.72 | -3.85 (-6.45 to -1.26) | 0.004 | 0.65 | -0.79 (-1.46 to -0.12) | 0.020 | 0.86 |
| Language IES | -2.49 (-3.47 to -1.52) | <0.001 | 0.73 | -4.27 (-6.74 to -1.79) | 0.001 | 0.70 | -0.95 (-1.6 to -0.31) | 0.004 | 0.89 |
| Memory IES | -1.08 (-1.69 to -0.47) | 0.001 | 0.84 | -5.17 (-7.73 to -2.61) | <0.001 | 0.73 | -0.91 (-1.6 to -0.22) | 0.010 | 0.87 |
| SDMT | -1.07 (-1.5 to -0.64) | <0.001 | 0.43 | -2.72 (-5.47 to 0.03) | 0.053 | 0.57 | -0.27 (-1 to 0.46) | 0.468 | 0.70 |
| Stroop colour | -1.1 (-1.52 to -0.67) | <0.001 | 0.43 | -2.14 (-4.98 to 0.7) | 0.138 | 0.53 | -0.38 (-1.09 to 0.32) | 0.283 | 0.75 |
| Stroop words | -1.17 (-1.61 to -0.74) | <0.001 | 0.40 | -2.16 (-4.94 to 0.62) | 0.126 | 0.54 | -0.32 (-1.02 to 0.39) | 0.376 | 0.73 |
| Stroop Interference | -0.78 (-1.2 to -0.35) | <0.001 | 0.48 | -4.13 (-6.86 to -1.39) | 0.003 | 0.63 | -0.59 (-1.33 to 0.15) | 0.119 | 0.75 |
| Verbal fluency | -0.72 (-1.1 to -0.34) | <0.001 | 0.61 | -3.98 (-6.71 to -1.25) | 0.005 | 0.63 | -0.62 (-1.34 to 0.11) | 0.093 | 0.77 |
| **Independence scale** |  |  |  |  |  |  |  |  |  |
| Global IES | -8.7 (-11.65 to -5.74) | <0.001 | 0.69 | -21.13 (-34.21 to -8.05) | 0.002 | 0.65 | -4.59 (-7.96 to -1.23) | 0.008 | 0.86 |
| Executive IES | -9.3 (-14.03 to -4.57) | <0.001 | 0.79 | -29.24 (-42.66 to -15.82) | <0.001 | 0.71 | -6.01 (-9.56 to -2.47) | 0.001 | 0.89 |
| Visual IES | -2.98 (-4.18 to -1.79) | <0.001 | 0.72 | -23.12 (-36.64 to -9.59) | 0.001 | 0.65 | -4.91 (-8.39 to -1.43) | 0.006 | 0.86 |
| Language IES | -13.32 (-18.44 to -8.2) | <0.001 | 0.73 | -25.78 (-38.78 to -12.78) | <0.001 | 0.70 | -5.83 (-9.22 to -2.45) | 0.001 | 0.89 |
| Memory IES | -6.18 (-9.36 to -3) | <0.001 | 0.84 | -30.18 (-43.5 to -16.85) | <0.001 | 0.73 | -5.53 (-9.12 to -1.94) | 0.003 | 0.87 |
| SDMT | -6.49 (-8.66 to -4.32) | <0.001 | 0.43 | -14.99 (-28.93 to -1.05) | 0.035 | 0.57 | -1.55 (-5.27 to 2.16) | 0.409 | 0.70 |
| Stroop colour | -6.05 (-8.27 to -3.84) | <0.001 | 0.43 | -13.66 (-28.44 to 1.11) | 0.07 | 0.53 | -2.65 (-6.32 to 1.02) | 0.155 | 0.75 |
| Stroop words | -6.23 (-8.53 to -3.93) | <0.001 | 0.40 | -14.65 (-29.27 to -0.03) | 0.05 | 0.54 | -2.45 (-6.16 to 1.26) | 0.192 | 0.73 |
| Stroop Interference | -4.45 (-6.68 to -2.22) | <0.001 | 0.48 | -24.21 (-38.47 to -9.95) | 0.001 | 0.63 | -3.67 (-7.53 to 0.2) | 0.063 | 0.75 |
| Verbal fluency | -4.63 (-6.55 to -2.72) | <0.001 | 0.61 | -21.76 (-35.66 to -7.85) | 0.002 | 0.63 | -3.48 (-7.16 to 0.21) | 0.064 | 0.77 |
| **Functional Checklist Score** | |  |  |  |  |  |  |  |  |
| Global IES | -2.9 (-3.85 to -1.95) | <0.001 | 0.69 | -6.69 (-10.9 to -2.48) | 0.002 | 0.65 | -0.63 (-1.71 to 0.45) | 0.252 | 0.86 |
| Executive IES | -3 (-4.53 to -1.46) | <0.001 | 0.79 | -9.47 (-13.83 to -5.11) | <0.001 | 0.71 | -1.11 (-2.26 to 0.04) | 0.059 | 0.89 |
| Visual IES | -1.06 (-1.44 to -0.68) | <0.001 | 0.72 | -7.05 (-11.34 to -2.77) | 0.001 | 0.65 | -0.69 (-1.79 to 0.41) | 0.218 | 0.86 |
| Language IES | -4.38 (-6.03 to -2.72) | <0.001 | 0.73 | -8.29 (-12.49 to -4.08) | <0.001 | 0.70 | -1.05 (-2.14 to 0.05) | 0.061 | 0.89 |
| Memory IES | -1.69 (-2.74 to -0.64) | 0.002 | 0.84 | -10.07 (-14.48 to -5.66) | <0.001 | 0.73 | -1.02 (-2.21 to 0.17) | 0.092 | 0.87 |
| SDMT | -1.68 (-2.43 to -0.93) | <0.001 | 0.43 | -6.23 (-11.03 to -1.44) | 0.011 | 0.57 | -0.01 (-1.29 to 1.27) | 0.987 | 0.70 |
| Stroop colour | -1.7 (-2.44 to -0.95) | <0.001 | 0.43 | -5.4 (-10.37 to -0.44) | 0.033 | 0.53 | -0.2 (-1.44 to 1.03) | 0.743 | 0.75 |
| Stroop words | -1.68 (-2.45 to -0.9) | <0.001 | 0.40 | -5.93 (-10.88 to -0.98) | 0.019 | 0.54 | -0.2 (-1.46 to 1.06) | 0.753 | 0.73 |
| Stroop Interference | -1.1 (-1.85 to -0.36) | 0.004 | 0.48 | -8.75 (-13.52 to -3.98) | <0.001 | 0.63 | -0.59 (-1.88 to 0.7) | 0.366 | 0.75 |
| Verbal fluency | -1.12 (-1.77 to -0.46) | 0.001 | 0.61 | -8.25 (-12.98 to -3.51) | 0.001 | 0.63 | -0.57 (-1.82 to 0.69) | 0.370 | 0.77 |

**exposure.**

*Results of linear models adjusted for cognitive test, Motor IES, antidepressant exposure before or at visit, age at visit, years of education. IES was normalized using the overall mean and standard deviation of all scores together by the measure of functional limitation and the other test scores were normalized using the mean and standard deviation of each score by the measure of functional limitation, then, normalized scores were inverted (multiplied by -1) for comparison with the IES scores.*

**Table S9. Association of normalized scores cognitive test and motor IES with and functional measures adjusted to for antipsychotics**

**exposure.**

|  | **Cognitive variable** | | | **Motor IES** | | | **Antipsychotics exposure** | | |
| --- | --- | --- | --- | --- | --- | --- | --- | --- | --- |
| **Total Functional Score** | **Coefficient (95%IC)** | **p-value** | **Tolerance** | **Coefficient (95%IC)** | **p-value** | **Tolerance** | **Coefficient (95%IC)** | **p-value** | **Tolerance** |
| Global IES | -1.5 (-2.08 to -0.91) | <0.001 | 0.66 | -3.39 (-5.93 to -0.86) | 0.009 | 0.64 | -0.75 (-1.4 to -0.11) | 0.023 | 0.73 |
| Executive IES | -1.53 (-2.44 to -0.63) | 0.001 | 0.78 | -4.65 (-7.24 to -2.06) | 0.001 | 0.69 | -1.09 (-1.75 to -0.43) | 0.001 | 0.79 |
| Visual IES | -0.51 (-0.74 to -0.27) | <0.001 | 0.67 | -3.78 (-6.39 to -1.18) | 0.005 | 0.65 | -0.78 (-1.46 to -0.11) | 0.023 | 0.72 |
| Language IES | -2.31 (-3.31 to -1.3) | <0.001 | 0.69 | -4.2 (-6.71 to -1.7) | 0.001 | 0.69 | -0.9 (-1.55 to -0.25) | 0.007 | 0.76 |
| Memory IES | -1.1 (-1.69 to -0.51) | <0.001 | 0.86 | -4.58 (-7.14 to -2.02) | 0.001 | 0.69 | -1.12 (-1.77 to -0.47) | 0.001 | 0.80 |
| SDMT | -1.02 (-1.46 to -0.59) | <0.001 | 0.41 | -2.66 (-5.4 to 0.08) | 0.057 | 0.57 | -0.4 (-1.13 to 0.33) | 0.279 | 0.60 |
| Stroop colour | -1.04 (-1.46 to -0.62) | <0.001 | 0.44 | -1.98 (-4.8 to 0.83) | 0.165 | 0.53 | -0.59 (-1.26 to 0.09) | 0.087 | 0.68 |
| Stroop words | -1.12 (-1.55 to -0.68) | <0.001 | 0.41 | -2.03 (-4.79 to 0.73) | 0.148 | 0.54 | -0.53 (-1.2 to 0.15) | 0.126 | 0.66 |
| Stroop Interference | -0.72 (-1.17 to -0.27) | 0.002 | 0.43 | -4.03 (-6.76 to -1.29) | 0.004 | 0.63 | -0.67 (-1.43 to 0.08) | 0.078 | 0.61 |
| Verbal fluency | -0.69 (-1.06 to -0.32) | <0.001 | 0.62 | -3.66 (-6.38 to -0.94) | 0.009 | 0.61 | -0.82 (-1.51 to -0.13) | 0.021 | 0.71 |
| **Independence scale** |  |  |  |  |  |  |  |  |  |
| Global IES | -8.11 (-11.11 to -5.11) | <0.001 | 0.66 | -19.97 (-32.97 to -6.96) | 0.003 | 0.64 | -5.28 (-8.6 to -1.97) | 0.002 | 0.73 |
| Executive IES | -8.66 (-13.3 to -4.01) | <0.001 | 0.78 | -26.54 (-39.84 to -13.24) | <0.001 | 0.69 | -7.06 (-10.45 to -3.67) | <0.001 | 0.79 |
| Visual IES | -2.7 (-3.92 to -1.47) | <0.001 | 0.67 | -22.25 (-35.71 to -8.79) | 0.001 | 0.65 | -5.5 (-8.98 to -2.03) | 0.002 | 0.72 |
| Language IES | -11.91 (-17.14 to -6.69) | <0.001 | 0.69 | -24.73 (-37.74 to -11.73) | <0.001 | 0.69 | -6.17 (-9.53 to -2.82) | <0.001 | 0.76 |
| Memory IES | -6.27 (-9.3 to -3.25) | <0.001 | 0.86 | -26.1 (-39.2 to -13) | <0.001 | 0.69 | -7.24 (-10.56 to -3.91) | <0.001 | 0.80 |
| SDMT | -6 (-8.21 to -3.8) | <0.001 | 0.41 | -14.64 (-28.45 to -0.84) | 0.038 | 0.57 | -2.98 (-6.65 to 0.68) | 0.11 | 0.60 |
| Stroop colour | -5.6 (-7.76 to -3.44) | <0.001 | 0.44 | -12.52 (-27.01 to 1.97) | 0.090 | 0.53 | -4.43 (-7.9 to -0.95) | 0.013 | 0.68 |
| Stroop words | -5.74 (-7.98 to -3.49) | <0.001 | 0.41 | -13.59 (-27.95 to 0.76) | 0.063 | 0.54 | -4.26 (-7.78 to -0.74) | 0.018 | 0.66 |
| Stroop Interference | -3.9 (-6.2 to -1.59) | 0.001 | 0.43 | -23.4 (-37.53 to -9.28) | 0.001 | 0.63 | -4.86 (-8.75 to -0.98) | 0.015 | 0.61 |
| Verbal fluency | -4.32 (-6.17 to -2.47) | <0.001 | 0.62 | -19.6 (-33.28 to -5.92) | 0.005 | 0.61 | -5.22 (-8.69 to -1.76) | 0.003 | 0.71 |
| **Funcitonal Checklist Score** |  |  |  |  |  |  |  |  |  |
| Global IES | -2.74 (-3.71 to -1.78) | <0.001 | 0.66 | -6.34 (-10.53 to -2.14) | 0.003 | 0.64 | -0.98 (-2.05 to 0.09) | 0.071 | 0.73 |
| Executive IES | -2.81 (-4.32 to -1.3) | <0.001 | 0.78 | -8.64 (-12.98 to -4.31) | <0.001 | 0.69 | -1.6 (-2.7 to -0.49) | 0.005 | 0.79 |
| Visual IES | -1 (-1.38 to -0.61) | <0.001 | 0.67 | -6.79 (-11.07 to -2.52) | 0.002 | 0.65 | -0.98 (-2.08 to 0.13) | 0.082 | 0.72 |
| Language IES | -4.06 (-5.74 to -2.37) | <0.001 | 0.69 | -7.93 (-12.13 to -3.73) | <0.001 | 0.69 | -1.28 (-2.36 to -0.2) | 0.021 | 0.76 |
| Memory IES | -1.66 (-2.68 to -0.65) | 0.001 | 0.86 | -8.88 (-13.27 to -4.5) | <0.001 | 0.69 | -1.71 (-2.82 to -0.6) | 0.003 | 0.80 |
| SDMT | -1.47 (-2.23 to -0.71) | <0.001 | 0.41 | -6.21 (-10.97 to -1.44) | 0.011 | 0.57 | -0.68 (-1.95 to 0.58) | 0.286 | 0.60 |
| Stroop colour | -1.51 (-2.24 to -0.78) | <0.001 | 0.44 | -5.21 (-10.11 to -0.3) | 0.038 | 0.53 | -0.95 (-2.13 to 0.23) | 0.113 | 0.68 |
| Stroop words | -1.47 (-2.23 to -0.7) | <0.001 | 0.41 | -5.74 (-10.64 to -0.85) | 0.022 | 0.54 | -0.96 (-2.16 to 0.25) | 0.118 | 0.66 |
| Stroop Interference | -0.89 (-1.67 to -0.12) | 0.024 | 0.43 | -8.5 (-13.22 to -3.77) | 0.001 | 0.63 | -1.2 (-2.5 to 0.11) | 0.071 | 0.61 |
| Verbal fluency | -0.98 (-1.62 to -0.35) | 0.003 | 0.619 | -7.65 (-12.34 to -2.96) | 0.002 | 0.61 | -1.28 (-2.47 to -0.1) | 0.034 | 0.71 |

*Results of linear models adjusted for cognitive test, Motor IES, antipsychotics exposure before or at visit, age at visit, years of education. IES was normalized using the overall mean and standard deviation of all scores together by the measure of functional limitation and the other test scores were normalized using the mean and standard deviation of each score by the measure of functional limitation, then, normalized scores were inverted (multiplied by -1) for comparison with the IES scores.*
